# Long non-coding RNA Malat1 fine-tunes bone homeostasis and repair by orchestrating cellular crosstalk and the β-catenin-OPG/Jagged1 pathway

**DOI:** 10.21203/rs.3.rs-3793919/v3

**Published:** 2024-10-11

**Authors:** Yongli Qin, Jumpei Shirakawa, Cheng Xu, Ruge Chen, Xu Yang, Courtney Ng, Shinichi Nakano, Mahmoud Elguindy, Zhonghao Deng, Kannanganattu V Prasanth, Moritz F. Eissmann, Shinichi Nakagawa, William M. Ricci, Baohong Zhao

**Affiliations:** 1 Arthritis and Tissue Degeneration Program and David Z. Rosensweig Genomics Research Center, Hospital for Special Surgery, New York, New York, USA.; 2 Department of Medicine, Weill Cornell Medical College, New York, New York, USA.; 3 Research Institute, Hospital for Special Surgery, New York, New York, USA.; 4 Department of Orthopaedic Surgery, Weill Cornell Medicine, New York, New York, USA.; 5 Department of Cell and Developmental Biology, Cancer center at Illinois, University of Illinois at Urbana-Champaign, Urbana, IL, USA; 6 Institute for Tumor Biology and Experimental Therapy, Paul-Ehrlich-Strasse 42-44, 60596 Frankfurt, Germany; 7 RNA Biology Laboratory, Faculty of Pharmaceutical Sciences, Hokkaido University, Sapporo 060-0812, Japan; 8 Orthopaedic Trauma Service, Hospital for Special Surgery & NewYork-Presbyterian Hospital, USA; 9 Graduate Program in Cell and Development Biology, Weill Cornell Graduate School of Medical Sciences, New York, New York, USA.

**Keywords:** Malat1, bone, fracture, lncRNA, long non-coding RNA, homeostasis, remodeling

## Abstract

The IncRNA Malat1 was initially believed to be dispensable for physiology due to the lack of observable phenotypes in Malat1 knockout (KO) mice. However, our study challenges this conclusion. We found that both Malat1 KO and conditional KO mice in the osteoblast lineage exhibit significant osteoporosis. Mechanistically, Malat1 acts as an intrinsic regulator in osteoblasts to promote osteogenesis. Interestingly, Malat1 does not directly affect osteoclastogenesis but inhibits osteoclastogenesis in a non-autonomous manner *in vivo* via integrating crosstalk between multiple cell types, including osteoblasts, osteoclasts and chondrocytes. Our findings substantiate the existence of a novel remodeling network in which Malat1 serves as a central regulator by binding to β-catenin and functioning through the β-catenin-OPG/Jagged1 pathway in osteoblasts and chondrocytes. In pathological conditions, Malat1 significantly promotes bone regeneration in fracture healing. Bone homeostasis and regeneration are crucial to well-being. Our discoveries establish a previous unrecognized paradigm model of Malat1 function in the skeletal system, providing novel mechanistic insights into how a lncRNA integrates cellular crosstalk and molecular networks to fine tune tissue homeostasis, remodeling and repair.

## Introduction

Recent genome-wide transcriptome analyses revealed that over 75% of the human genome is transcribed, among which about 95% are transcripts without coding capacity. Long noncoding RNAs (lncRNAs) emerge as a relatively new category of non-coding RNAs (≥ 500 nucleotides ^1^). It is now widely accepted that lncRNAs can perform diverse regulatory roles in regulation of gene expression. lncRNAs are enriched in the nucleus and/or cytoplasm and possess the ability to interact with versatile biomolecules, including various proteins, chromosomal DNAs and RNAs. Therefore, in contrast to miRNAs, lncRNAs regulate gene expression and function by diverse mechanisms, such as functioning as scaffolds for transcriptional and chromatin-modifying complex assemblies, as enhancers or decoys regulating gene transcription, and as cis-acting or trans-acting regulators involved in gene expression and epigenetic regulation ^2–5^. Importantly, lncRNAs are druggable targets, and identification of the functional importance of lncRNAs has unveiled new diagnostic and therapeutic opportunities for human diseases, such as cancer, cardiovascular diseases and genetic disorders ^2,6–13^. Understanding the biological importance and clinical relevance of lncRNAs is at the forefront of RNA biology research. Nonetheless, only a small fraction of lncRNAs have well-established identifications. Most lncRNAs lack functional annotations, particularly with genetic evidence in vivo and convincing mechanistic studies.

Malat1 (metastasis associated lung adenocarcinoma transcript 1) is a highly evolutionarily conserved and abundant nuclear lncRNA. It is a 6.8 kilo-nucleotide RNA Polymerase II transcript from mouse chromosome 19 (8.9 kilo-nucleotide from human chromosome 11). As one of the first discovered lncRNAs, Malat1 was initially recognized as a gene showing specific upregulation in metastatic non-small-cell lung cancer cells^14^. Subsequent studies found a variety of associations between Malat1 and the growth and metastasis of different cancers, such as lung cancer, hepatocellular carcinoma, and breast cancer ^15,16^. In physiological settings, although Malat1 was reported to be a key component of nuclear speckle and a regulator of alternative pre-mRNA splicing in *in vitro* studies, Malat1 knockout (KO) mice surprisingly appeared to have no defects in nuclear speckle assembly or pre-mRNA splicing ^17–21^, raising questions about the extent to which Malat1 contributes to these processes *in vivo*. These unexpected discrepancies of *in vitro* and in vivo studies also draw increasing attention on the importance and necessity of *in vivo* studies to reveal lncRNA function. The homeostatic/physiological function of Malat1 *in vivo* has continued to be an enigma, because the absence of this abundant lncRNA in mice does not seem to exhibit abnormalities ^17–19^. However, to the best of our knowledge, there are no studies that have comprehensively characterized bone phenotype of Malat1 KO mice.

Bone is a vital organ, which plays a fundamental role in providing structural support to the body, enabling movement, and protecting many other organs. Bone homeostasis is crucial to the quality of life and overall well-being. Bone homeostasis in adulthood is mainly maintained by an active bone remodeling process, which requires a delicate balance between osteoclast-mediated bone resorption and osteoblast-mediated bone formation. Bone tissues undergo constant remodeling, during which bone resorption and formation are usually coupled to ensure that osteoclast-generated resorption lacunae are filled with new bone produced by osteoblasts. This coordination helps maintain bone homeostasis and also provides a mechanism for adapting the skeleton to environmental changes and repairing bone damage. There exists a variety of crosstalk between two major bone cell types, bone-resorbing osteoclasts and bone-forming osteoblasts, as well as other cells. The intricate cellular crosstalk coordinately couples the activities of different cells to maintain bone homeostasis during remodeling ^22,23^. In pathological conditions, bone remodeling is often deregulated, which results in unbalanced bone resorption and formation. For example, excessive osteoclast formation accompanied by extensive bone resorption but with limited bone formation/repair often occurs in rheumatoid arthritis (RA), periodontitis and osteoporosis. On the other hand, excessive bone formation and/or defective bone resorption result in osteopetrosis or osteosclerosis. Notably, unbalanced activities between osteoclasts and osteoblasts in pathological bone remodeling synergize to aggravate the rate and extent of bone damage ^24–27^. Although the importance of bone remodeling is evident to bone homeostasis and health, the coupling and crosstalk mechanisms are complex and far from well understood.

Given the vital importance of bone remodeling to skeletal health and overall well-being, we took advantage of rigorous genetic approaches using global and conditional Malat1 KO mice in this study. Contrary to previously held beliefs that Malat1 had no appreciable phenotype, we uncovered that Malat1 KO mice exhibit a significant osteoporotic bone phenotype characterized by reduced osteoblastic bone formation and enhanced osteoclastic bone resorption *in vivo*. Thus, Malat1 deletion uncoupled the normal bone remodeling process between osteoblasts and osteoclasts. Our data further demonstrate that Malat1 emerges as a novel regulator impacting multiple cell types, including osteoblasts, osteoclasts, and chondrocytes, through the β-catenin-OPG/Jagged1 pathway. This study discovered an important homeostatic function of Malat1, and identified this lncRNA as a previously unrecognized key bone remodeling regulator that controls both bone formation and resorption to maintain bone homeostasis, regeneration and health.

## Results

### Malat1 is a novel lncRNA regulator of bone homeostasis and remodeling

**Fig. 1:**
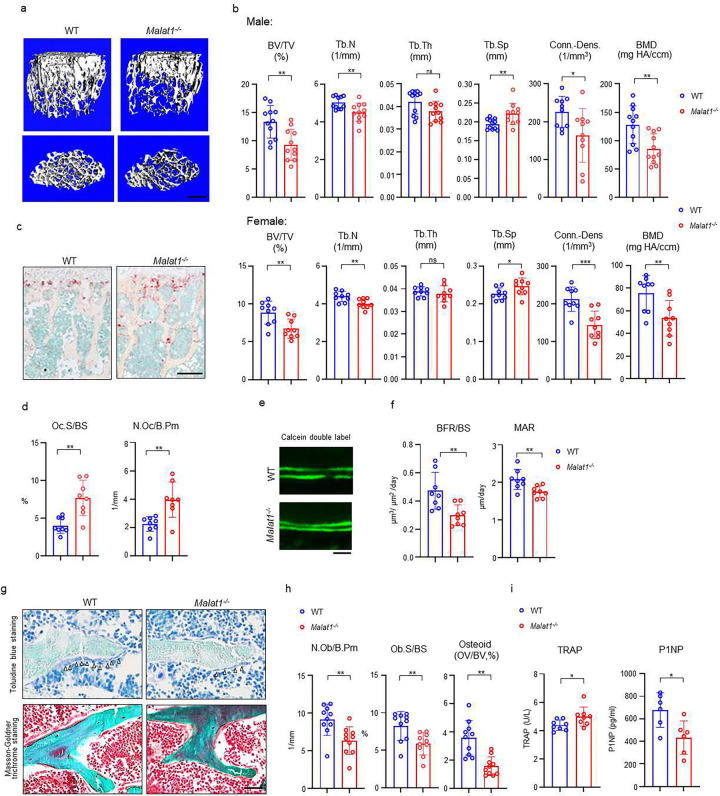
Malat1 deficiency disrupts bone remodeling and results in osteoporosis through reduced osteoblastic bone formation and increased osteoclastic bone resorption. (**a**) μCT images and (**b**) bone morphometric analysis of trabecular bone of the distal femurs isolated from the 12-week-old-male (n=11, upper panel) and female (n=9, lower panel) WT and *Malat1*^*−/−*^ littermate mice. BV/TV, bone volume per tissue volume; BMD, bone mineral density; Conn-Dens., connectivity density; Tb.N, trabecular number; Tb.Th, trabecular thickness; Tb.Sp, trabecular separation. (**c**) TRAP staining and (**d**) histomorphometric analysis of histological sections obtained from of 12-week-old male WT and *Malat1*^*−/−*^ littermate mice (n = 8/group). Oc.S/BS, osteoclast surface per bone surface; N.Oc/B.Pm, number of osteoclasts per bone perimeter. (**e**) Images of calcein double labelling of the tibia of 12-week-old male WT and *Malat1*^*−/−*^ littermate mice. (**f**) Dynamic histomorphometric analysis of mineral apposition rate (MAR) and bone formation rate per bone surface (BFR/BS) after calcein double labeling of the tibiae of WT and *Malat1*^*−/−*^ littermate male mice (n = 8/group). (**g**) Representative images of Toluidine blue staining (top) and Masson-Goldner staining (bottom) of femur from 12-week-old-male WT and *Malat1*^*−/−*^ littermate mice. For Toluidine blue staining, the bones show green and osteoblasts are indicated by arrow heads. For Masson-Goldner staining, osteoid matrix appears dark orange on the surface of the bone beneath the osteoblasts (indicated by dash lines), osteoblasts are stained orange lining on the bone surface, and bone marrow cells appear red in the photograph. (**h**) Bone morphometric analysis of osteoblast surface per bone surface (Ob.S/BS), osteoblast number per bone perimeter (N.Ob/B.Pm) and osteoid matrix volume per bone volume (OV/BV) of the femur of WT and *Malat1*^*−/−*^ littermate male mice (n = 10/group). (**i**) serum TRAP and P1NP levels of 12-week-old male mice. b, d, f, h, i *p < 0.05; **p < 0.01; ns, not statistically significant by Student’s t test. Data are mean ± SD. Scale bars: a 400 μm; c 200 μm; e, g 50 μm.

We first performed a series of experiments to comprehensively examine bone mass, bone resorption, bone formation, bone cell changes and bone remodeling by microCT (μCT), bone histology and dynamic histomorphometric analysis. Adult mice with Malat1 deficiency (*Malat1*^*−/−*^ on C57BL/6 background) do not show growth retardation or macroscopic differences (Suppl. Fig. 1a), but exhibit significantly reduced bone mass compared with wild type littermate controls (WT) (Fig.1a). μCT analysis showed markedly decreased trabecular bone volume (BV/TV), number (Tb.N.), bone mineral density (BMD) and connectivity density (Conn-Dens.), and increased trabecular spacing (Tb. Sp) in both male and female *Malat1*^*−/−*^ mice (Fig.1b). Cortical bone appears normal (Suppl. Fig. 1b). Osteoclastic bone resorption was significantly enhanced in *Malat1*^*−/−*^ mice indicated by the elevated numbers and surfaces of osteoclasts (Fig. 1c, d). However, osteoblastic bone formation was not accordingly enhanced but instead was dramatically suppressed by Malat1 deficiency (Fig. 1e–h). Bone dynamic histomorphometric analysis by calcein double labeling showed that Malat1 absence notably inhibited both mineral apposition rate (MAR) and bone formation rate (BFR/BS) in *Malat1*^*−/−*^ mice (Fig. 1e, f). Furthermore, osteoblast parameters, such as osteoblast surfaces and numbers, as well as osteoid formed beneath osteoblasts were significantly reduced in *Malat1*^*−/−*^ mice (Fig.1g, h). The serum osteoblastic bone formation marker P1NP was decreased, while osteoclastic bone resorption marker TRAP was increased, in *Malat1*^*−/−*^ mice (Fig. 1i). These results indicate that the lack of Malat1 suppresses osteogenesis and bone formation in mice. Moreover, Malat1 deficiency uncouples osteoclastic bone resorption and osteoblastic bone formation, leading to markedly reduced bone mass. Our data thus identify Malat1 as a novel bone remodeling regulator in bone homeostasis.

### Malat1 acts as a cell-intrinsic regulator in osteoblasts to promote bone formation

**Fig. 2:**
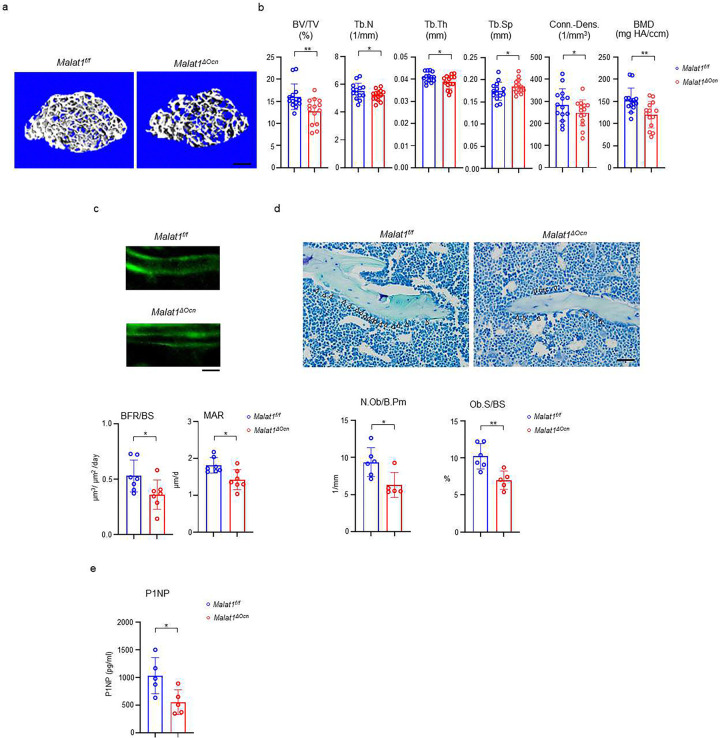
Specific deletion of Malat1 in osteoblasts leads to reduced bone mass and defects in bone formation. (**a**) μCT images and (**b**) bone morphometric analysis of trabecular bone of the distal femurs isolated from the 12-week-old male *Malat1*^*f/f*^ and *Malat1*^*ΔOcn*^ littermate mice (n = 14/group). (**c**) Images of calcein double labelling (top) of the tibia of 12-week-old male *Malat1*^*f/f*^ and *Malat1*^*ΔOcn*^ littermate mice. Dynamic histomorphometric analysis (bottom) of mineral apposition rate (MAR) and bone formation rate per bone surface (BFR/BS) after calcein double labeling of the tibiae of *Malat1*^*f/f*^ and *Malat1*^*ΔOcn*^ littermate male mice (n = 7/group). (**d**) Representative images of Toluidine blue staining (top) of femur from 12-week-old male *Malat1*^*f/f*^ and *Malat1*^*ΔOcn*^ littermate mice. For Toluidine blue staining, the bones show green and osteoblasts are indicated by arrow heads. Bone morphometric analysis (bottom) of osteoblast surface per bone surface (Ob.S/BS) and osteoblast number per bone perimeter (N.Ob/B.Pm) of the femur of 12-week-old male *Malat1*^*f/f*^ and *Malat1*^*ΔOcn*^ littermate mice. (e) serum P1NP levels of 12-week-old male mice. b, c, d, e *p < 0.05; **p < 0.01 by Student’s t test; ns, not statistically significant. Data are mean ± SD. Scale bars: a 200 μm; c, d 50 μm.

Next, we examined the role of Malat1 in osteoblastic bone formation. We crossed *Malat1*^*flox/flox*^ mice with the mice expressing Cre under the control of *Osteocalcin (Ocn)* promoter to generate Malat1 conditional KO mice specifically in osteoblasts (*Malat1*^*flox/flox*^*OcnCre(+);* hereafter referred to as *Malat1*^*ΔOcn*^). Malat1 deficiency in osteoblasts significantly decreased trabecular bone volume (BV/TV), number (Tb.N.), thickness (Tb. Th), bone mineral density (BMD) and connectivity density (Conn-Dens.), and increased trabecular spacing (Tb. Sp) in *Malat1*^*ΔOcn*^ mice (Fig. 2a, b). There are no obvious changes in cortical bone phenotype (Suppl. Fig. 2). Furthermore, mineral apposition rate (MAR) and bone formation rate (BFR/BS) were both lower in *Malat1*^*ΔOcn*^ mice than the controls (Fig. 2c). In parallel, Malat1 deficiency in osteoblasts led to decreased osteoblast numbers and surfaces in vivo (Fig. 2d). The serum osteoblastic bone formation marker P1NP was decreased in *Malat1*^*ΔOcn*^ mice (Fig. 2e). These results indicate that *Malat1*^*ΔOcn*^ mice exhibit osteoporotic phenotype with reduced bone formation, which is consistent with *Malat1*^*−/−*^ mice. Therefore, Malat1 is a cell-intrinsic osteogenic regulator that promotes osteoblastic bone formation.

### Malat1 binds to β-catenin and suppresses its transcriptional activity in osteoblasts

**Fig. 3:**
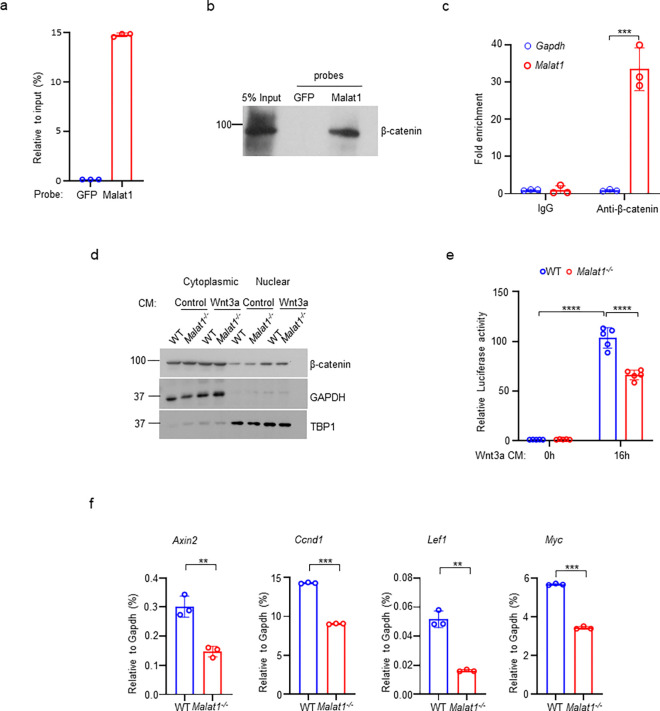
Malat1 binds to β-catenin to positively regulate canonical Wnt/ β-catenin signaling pathway. (**a**) ChIRP analysis of the specificity and efficiency of the Malat1 probe. Mouse Malat1 or the control GFP probes were used to pull down endogenous *Malat1* from MC3T3-E1 cells, followed by qPCR quantification of Malat1. (**b**) ChIRP analysis of the Malat1 binding to β-catenin. Mouse Malat1-specific probes were used to pull down the endogenous Malat1 in the MC3T3-E1 cells, followed by immunoblotting with anti-β-catenin antibody. (**c**) RIP assay of β-catenin binding to Malat1. Endogenous β-catenin was immunoprecipitated from MC3T3-E1 cells, and the β-catenin-bound Malat1 was quantitated by qPCR. Rabbit IgG was used as a negative control IP antibody. (**d**) Immunoblot analysis of the nuclear and cytoplasmic localization of β-catenin in calvarial osteoblasts that were serum starved for 16h, followed by treatment with 50% Wnt3a- or the control L- conditional medium for 1h. TBP1 and GAPDH were measured as loading controls for nuclear and cytoplasmic fractions, respectively. Experiments in a-d were replicated three times. (**e**) Luciferase reporter assay of the Wnt/β-catenin signaling activity measured from the indicated calvarial osteoblasts transfected with the M50 Super 8x TOPFlash reporter plasmid and pRL-Tk control plasmid for 48 h, followed by treatment with or without 20% Wnt3a conditional medium for 16h (n = 5). (**f**) qPCR analysis of mRNA expression of β-catenin target genes in calvarial osteoblasts in the osteogenic medium (α-MEM with 10% FBS supplemented with 10mM β-glycerophosphate and 100 ug/ml ascorbic acid) for seven days (n=3). Data are mean ± SD. c,e ***p < 0.001; ****p < 0.0001 by two-way ANOVA with Bonferroni’s multiple comparisons test. f, **p < 0.01; ***p < 0.001 by Student’s t test.

Given these findings, we sought to investigate the mechanisms underlying the regulation of osteoblastic bone formation by Malat1. β-catenin is a central transcriptional factor in canonical Wnt signaling pathway, and plays an important role in positively regulating osteoblast differentiation and function ^28–33^. Upon stimulation, most notably from canonical Wnt ligands, β-catenin is stabilized and translocates into the nucleus, where it interacts with coactivators to activate target gene transcription. Previous reports observed a link between Malat1 and β-catenin signaling pathway in cancers ^34,35^, but the underlying molecular mechanisms in terms of how Malat1 interacts with β-catenin and regulates its nuclear retention and transcriptional activity are unclear. In this study, we performed both Chromatin isolation by RNA purification (ChIRP) assay (Fig. 3a, b, Supplementary Fig. 3) and RNA immunoprecipitation (RIP) assay (Fig. 3c) to determine the interaction between Malat1 and β-catenin. The results obtained by these two approaches clearly show that Malat1 binds to β-catenin in osteoblasts (Fig. 3a–c). Next, we asked whether Malat1 regulates β-catenin nuclear translocation in response to Wnt3a. We did not find that Malat1 deficiency significantly affects nuclear localization of β-catenin stimulated by Wnt3a (Fig. 3d, Supplementary Fig. 4). We then performed Luciferase assay to examine whether Malat1 modulates the transcriptional activity of β-catenin. The results showed that Malat1 deficiency significantly reduced the transcriptional activity of β-catenin in response to Wnt3a stimulation (Fig. 3e). In line with this, the expression levels of β-catenin target genes, such as *Axin2, Ccnd1, Lef1 and Myc*, were substantially lower in *Malat1*^*−/−*^ cells than WT controls (Fig. 3f). Our findings indicate that Malat1 is a key regulator of Wnt/β-catenin signaling pathway that is important for osteoblasts. Since Malat1 is a nuclear lncRNA, these data also suggest that Malat1 acts as a scaffold to tether β-catenin in nuclei to implement its positive regulation of β-catenin activity.

### Malat1 inhibits osteoclastogenesis in a non-autonomous manner

**Fig. 4:**
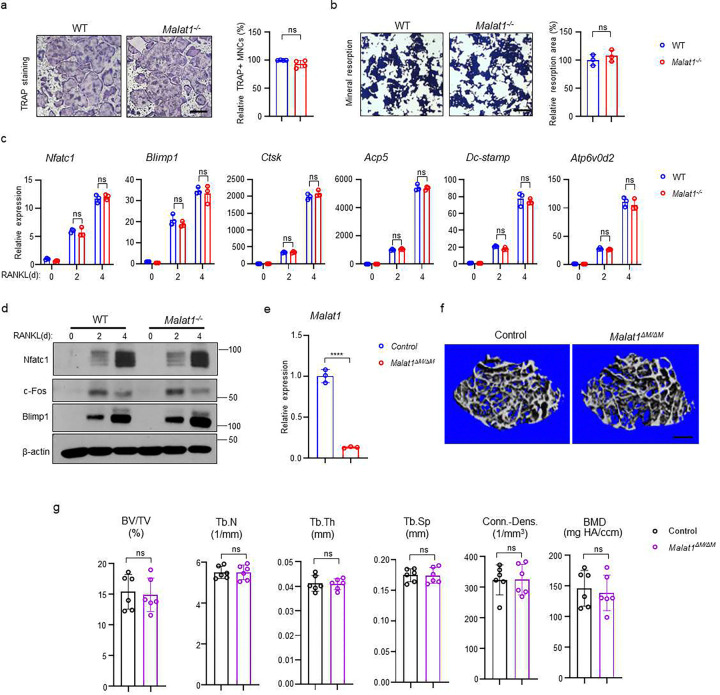
Malat1 is not an intrinsic regulator of osteoclast differentiation. (**a**) Osteoclast differentiation using BMMs obtained from WT and *Malat1*^*−/−*^ mice stimulated with RANKL for 3 days. TRAP staining (left panel) was performed and the area of TRAP-positive MNCs (≥3 nuclei/cell) per well relative to the WT control was calculated (right panel). (n =4/group). (**b**) Von Kossa staining (left) and the resorption area (%) (right) of the osteoclast cultures of WT and *Malat1*^*−/−*^ BMMs stimulated with RANKL for 4 days. (n = 3/group). Mineralized area: black; resorption area: white. (**c**) qPCR analysis of mRNA expression of the indicated genes during osteoclastogenesis with or without RANKL for 2 days and 4 days. (**d**) Immunoblot analysis of Nfatc1, Blimp1 and c-Fos expression during osteoclastogenesis with or without RANKL for 2 days and 4 days. β-actin was used as a loading control. (**e-g**) Malat1 deletion efficiency (**e**) and μCT images (**f**) and bone morphometric analysis (**g**) of trabecular bone of the distal femurs isolated from the indicated 12-week-old male Control and *Malat1*^*ΔM/ ΔM*^ littermate mice (n = 6/group). Data are mean ± SD. a,b,f ns, not statistically significant by Student’s t test; c, by two-way ANOVA with Bonferroni’s multiple comparisons test. Scale bars: a,b 100 μm; e 400 μm

As osteoclast formation is significantly increased in *Malat1*^*−/−*^ mice, we examined whether this osteoclast change is due to an intrinsic role of Malat1 in these cells. We first investigated *in vitro* osteoclast differentiation using bone marrow derived macrophages (BMMs) as osteoclast precursors. Surprisingly, there is no significant difference in osteoclast differentiation and mineral resorption between WT and *Malat1*^*−/−*^ BMM cultures (Fig. 4a, b), which is inconsistent with the *in vivo* data (Fig. 1c). Moreover, the expression of osteoclastogenic transcription factors and osteoclast marker genes was similar between WT and *Malat1*^*−/−*^ cultures (Fig. 4c, d). We also generated *Malat1*^*flox/flox*^*LysMCre(+)* (*Malat1*^*ΔM/ ΔM*^) mice, a myeloid lineage osteoclast specific Malat1 conditional KO mouse line. The deletion efficiency of Malat1 in the LysM-Cre mice is very high (>90%) (Fig. 1e). However, there is no significant difference in bone mass between *Malat1*^*ΔM/ ΔM*^ mice and their littermate control *LysMCre(+)* mice (Fig. 4f, g). These results demonstrate that Malat1 expressed in osteoclastic lineage cells does not affect osteoclastogenesis. Therefore, Malat1 inhibits osteoclastogenesis in a non-autonomous manner *in vivo*.

### Malat1 couples osteoblast-osteoclast crosstalk via β-catenin-OPG axis

**Fig. 5:**
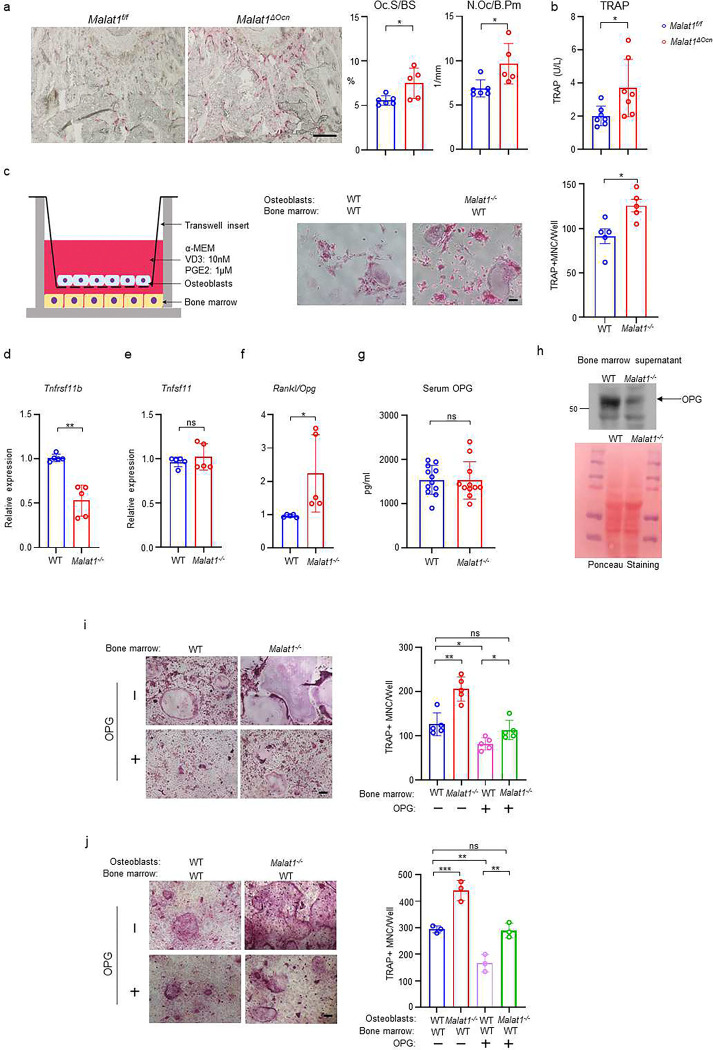
Malat1 promotes OPG expression in osteoblasts to suppress osteoclastogenesis. (**a**) TRAP staining (left) and histomorphometric analysis (right) of histological sections obtained from the metaphysis region of distal femurs from the 12-week-old male *Malat1*^*f/f*^ and *Malat1*^*ΔOcn*^ littermate mice. n = 5–6/group. Oc.S/BS, osteoclast surface per bone surface; N.Oc/B.Pm, number of osteoclasts per bone perimeter. (b) serum TRAP levels of 12-week-old male mice. (**c**) A schematic diagram (left) of the co-culture system with primary osteoblasts and bone marrow cells in trans-wells. TRAP staining (middle) was performed and the number of TRAP-positive MNCs (≥3 nuclei/cell) per well was calculated (right panel). (n =5 replicates from two experiments). (**d-e**) qPCR analysis of mRNA expression of *Tnfrsf11b* (encoding OPG) (**d**) and *Tnfsf11* (encoding RANKL) (**e**) in calvarial osteoblasts (n =5/group). (**f**) The expression ratio of Rankl/Opg in calvarial osteoblasts. (**g**) ELISA analysis of OPG levels in the serum from the 12-week-old male WT and *Malat1*^*−/−*^ mice (n = 11–12/group). (**h**) Immunoblot analysis of OPG expression in the bone marrow supernatant from the 12-week-old male WT and *Malat1*^*−/−*^ mice. Bottom: Ponceau Staining of the gels showing an equivalent amount of total proteins loaded between samples. (**i**) Osteoclast differentiation of WT and *Malat1*^*−/−*^ bone marrows stimulated with RANKL (40 ng/ml) and M-CSF C.M. (1:20) with or without OPG (2.5ng/ml) for five days. TRAP staining (left panel) was performed and the number of TRAP-positive MNCs (≥3 nuclei/cell) per well was calculated (right panel). TRAP-positive cells appear red in the photographs. n = 5 replicates. (**j**) Osteoclast differentiation of the cocultures of the indicated calvarial osteoblasts and WT bone marrow cells treated with 10 nM of VitD3 and 1 μM of prostaglandinE2 for 6 days in the presence or absence of OPG (1ng/ml). TRAP staining (left) was performed and the number of TRAP-positive MNCs (≥3 nuclei/cell) per well was calculated (right panel). n = 3 replicates. Data are mean ± SD. a-g, *p < 0.05; **p < 0.01 by Student’s t test; i,j *p < 0.05, **p < 0.01, ***p < 0.001 by two-way ANOVA with Bonferroni’s multiple comparisons test . ns, not statistically significant. Scale bars: a 200 μm; c,i,j 100 μm

It is intriguing how Malat1 regulates osteoclastogenesis. We first looked at osteoclast phenotype in *Malat1*^*ΔOcn*^ mice, and found that Malat1 deletion in osteoblasts significantly enhanced osteoclast numbers and surfaces *in vivo* (Fig. 5a). The serum osteoclastic bone resorption marker TRAP was increased in *Malat1*^*ΔOcn*^ mice (Fig. 5b). This finding indicates that Malat1 affects osteoclast formation through crosstalk between osteoblasts and osteoclasts. To further explore the underlying mechanisms of this crosstalk, we took advantage of a well-established co-culture system ^36^ with primary osteoblasts in a transwell and bone marrow cells on the bottom of the dish. RANKL and M-CSF secreted by osteoblasts induced osteoclast differentiation on the bottom (Fig. 5c). Interestingly, we found that more osteoclasts formed in the co-cultures with *Malat1*^*−/−*^ osteoblasts than WT osteoblasts (Fig. 5c). The results of this co-culture system recapitulate the *in vivo* enhanced osteoclast phenotype, and indicates that certain soluble factors secreted from osteoblasts are highly likely involved in the Malat1 regulation of osteoclastogenesis. Since we found that Malat1 binds to β-catenin to regulate its target genes, we primarily searched β-catenin target genes that function as secreted factors. Osteoprotegerin (OPG), encoded by *Tnfrsf11b*, is a β-catenin target ^37,38^. OPG is a secreted factor that acts as a RANKL decoy receptor to block RANKL activity, thereby suppressing osteoclast formation and bone resorption ^39^. We found that OPG expression was markedly lower in the *Malat1*^*−/−*^ osteoblasts than the WT control cells (Fig. 5d). RANKL-OPG axis is a typical bone remodeling mediator through impacting osteoclastogenesis, and RANKL/OPG ratio is usually an indicator for osteoclastogenic/bone resorption potential and status ^40^. Our results showed that OPG but not RANKL was down-regulated in *Malat1*^*−/−*^ osteoblasts, leading to a higher RANKL/OPG ratio in *Malat1*^*−/−*^ osteoblasts than WT controls (Fig. 5e, f). The elevated RANKL/OPG ratio in *Malat1*^*−/−*^ osteoblasts favors enhanced osteoclastogenesis, which is aligned with the enhanced osteoclast formation and decreased bone mass in *Malat1*^*−/−*^ mice. We next examined the OPG expression *in vivo*. Recent literature demonstrates that locally produced OPG in bone, but not the serum OPG, is a critical inhibitor of osteoclast formation and bone resorption ^41^. Indeed, the OPG level in serum in *Malat1*^*−/−*^ mice is similar to that in WT controls (Fig. 5g). However, OPG level in bone marrow was drastically decreased in *Malat1*^*−/−*^ mice compared to WT mice (Fig. 5h). We further tested the importance of the decreased OPG level in the Malat1 regulation of osteoclasts. We applied two well-established *ex vivo* culture systems to closely recapitulate in vivo conditions. In the whole bone marrow cultures with M-CSF and RANKL (Fig. 5i), Malat1 deletion in *Malat1*^*−/−*^ bone marrow enabled more osteoclastogenesis than WT bone marrow, which is consistent with the *in vivo* findings. When recombinant OPG was added to the bone marrow cultures, the osteoclast formation in both WT and *Malat1*^*−/−*^ cultures was inhibited as expected, but the osteoclast formation in *Malat1*^*−/−*^ bone marrow with additional OPG became similar to that in the WT control cultures without additional OPG (Figure 5i column 1 vs 4). In another co-culture system (Fig. 5j), WT or *Malat1*^*−/−*^ osteoblasts were co-cultured with WT bone marrow in the presence of 1,25(OH)_2_-vitD3 and PGE2. The RANKL secreted from osteoblasts induces osteoclast formation in this co-culture system. Furthermore, because these cultures included the same WT bone marrow cells but different osteoblasts from WT or *Malat1*^*−/−*^ mice, the results directly reflected osteoblastic Malat1 effects on osteoclast formation. In this co-culture system, we observed a similar phenotype as that in Fig. 5i. Extra OPG can lead to osteoclast formation in *Malat1*^*−/−*^ osteoblasts and WT bone marrow cocultures comparable to that in WT osteoblasts and WT bone marrow cocultures without extra OPG (Fig. 5j column 1 vs 4). These results collectively support that the decreased OPG level in *Malat1* deficient osteoblasts plays a significant role in the excessive osteoclast formation in *Malat1*^*−/−*^ mice (Fig. 1c, d) and *Malat1*^*ΔOcn*^ mice (Fig. 5a), as illustrated in Fig. 7.

### Malat1 positively regulates the production of β-catenin targets, OPG and Jagged1, from chondrocytes

**Fig. 6:**
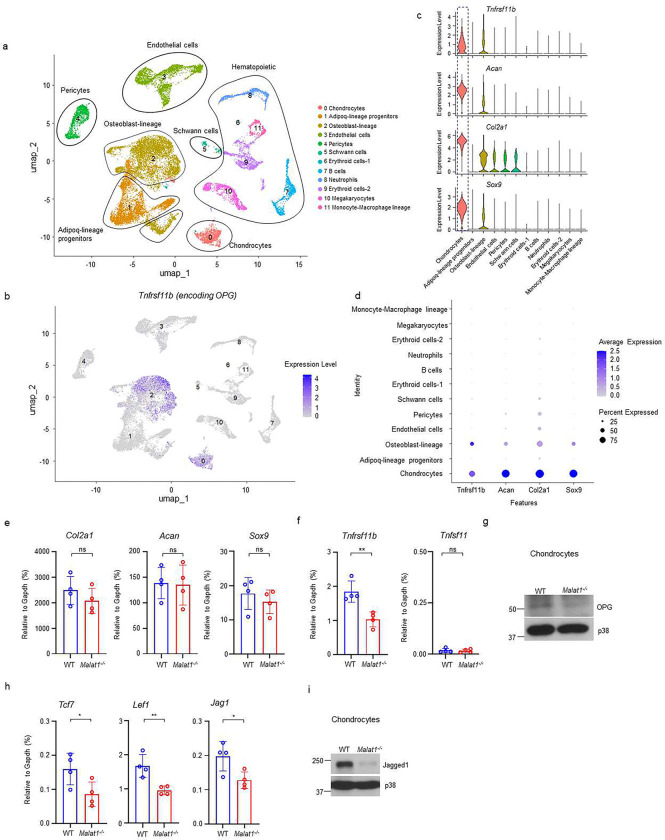
Malat1 enhances OPG and Jagged1 expression in chondrocytes. **(a)** UMAP plot analysis of the bone and bone marrow datasets of scRNAseq based on GSE128423. (**b**) UMAP plot of the expression of *Tnfrsf11b* (encoding OPG) in bone and bone marrow cells. (**c**) Violin plots of the expression of *Tnfrsf11b, Acan, Col2a1* and *Sox9*. (**d**) Dot plot of the expression of *Tnfrsf11b, Acan, Col2a1* and *Sox9* across the listed scRNAseq clusters. Cell clusters are listed on y-axis. Features are listed along the x-axis. Dot size reflects the percentage of cells in a cluster expressing each gene. Dot color reflects the scaled average gene expression level as indicated by the legend. (**e, f, h**) qPCR analysis of the indicated genes in primary chondrocytes. n = 4/group. (**g, i**) Immunoblot analysis of OPG and Jagged1 in the chondrocytes isolated from the WT and *Malat1*^*−/−*^ mice. Data are mean ± SD. e,f,h, *p < 0.05; **p < 0.01 by Student’s t test; ns, not statistically significant.

As OPG is a key osteoclastic inhibitor, we asked whether other cells, in addition to osteoblasts, in bone marrow could also produce OPG, which we have established is regulated by Malat1. We analyzed a bone scRNAseq dataset (GSE128423) ^42^. Except for osteoblasts as expected, we surprisingly found that chondrocytes express a high level of OPG in the analyzed cells from bone (Fig. 6a–d, Supplementary Fig. 5). We then isolated primary chondrocytes from mouse knees, which highly express chondrocyte marker genes, such as *Sox9, Acan and Col2a1* (Fig. 6e, Supplementary Fig. 6b). These cells were also Alcian blue positive (Supplementary Fig. 6a). We confirmed OPG expression in chondrocytes isolated from mice (Fig. 6g). Moreover, OPG expression level in *Malat1*^*−/−*^ chondrocytes was approximately 50% less compared to that in WT cells (Fig. 6f, g). RANKL (encoded by *Tnfsf11*) is nearly undetectable in chondrocytes (Fig. 6f). The chondrocyte marker genes, including *Sox9, Acan and Col2a1,* were not affected by Malat1 (Fig. 6e). These data show that chondrocytes are an important cellular source of OPG highly expressed in bone, which is critically regulated by Malat1.

**Fig. 7:**
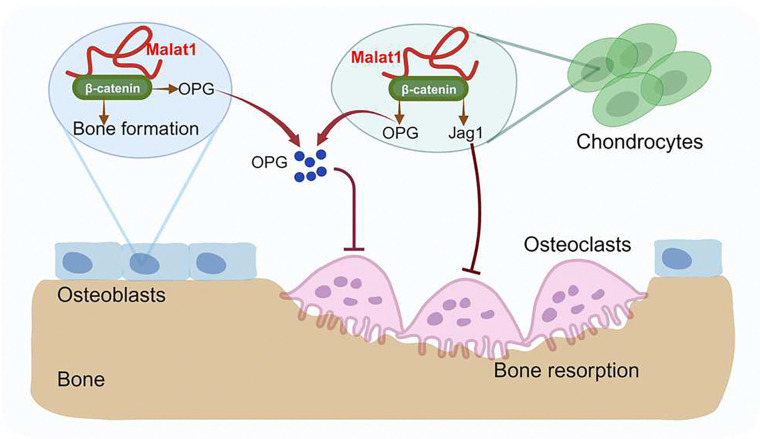
A model illustrating a Malat1-centered molecular and cellular network in bone remodeling. Malat1 binds to β-catenin, regulating its transcriptional activity on downstream target genes, such as *Tnfrsf11b* (encoding OPG) and *Jag1* (encoding Jagged1), both of which are osteoclastogenic inhibitors. Malat1 orchestrates β-catenin to promote intrinsic osteoblastic bone formation while suppressing osteoclastogenesis in a non-autonomous manner through β-catenin target genes OPG and Jagged1, expressed by osteoblasts and chondrocytes.

We next examined whether Malat1 also modulates β-catenin activity in chondrocytes. In addition to OPG, we observed that the expression of other β-catenin target genes, such as *Tcf7*, *Lef1* and *Jag1*, was approximately 50% lower in *Malat1*^*−/−*^ chondrocytes than that in WT cells (Fig. 6h). This suggests that, similar to osteoblasts, Malat1 positively regulates β-catenin activity in chondrocytes. The decreased expression of Jagged1 in *Malat1*^*−/−*^ chondrocytes drew our attention, as Jagged1 is not only a Notch ligand ^43^ but also an inhibitor of osteoclastogenesis ^44,45^. Following this observation, we evaluated the protein expression levels of Jagged1, and found that Malat1 deficiency drastically decreased Jagged1 protein expression in chondrocytes (Fig. 6i). These results collectively indicate that the decreased OPG and Jagged1 production in *Malat1*^*−/−*^ chondrocytes also contributes to the enhanced osteoclast formation and low bone mass in *Malat1*^*−/−*^ mice. Thus, Malat1 links the activities of chondrocytes and osteoclasts through β-catenin-OPG/Jagged1 axis (Fig. 7).

### Malat1 promotes bone regeneration in fracture healing

**Fig. 8:**
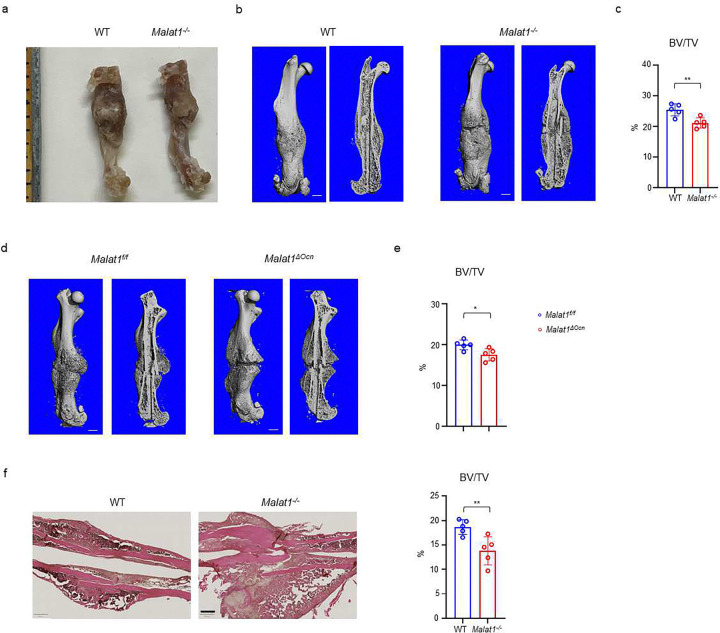
Malat1 enhances bone regeneration in fracture healing. (**a**) Representative photograph of femur fracture callus. (**b, d**) representative μCT images of femurs isolated from the indicated mice at day 21 post-fracture. (**c, e**) μCT analysis of BV/TV in callus area of femurs of the indicated mice at day 21 post-fracture. (**f**) HE staining and histological analysis of the callus areas. n=5/group. Data are mean ± SD. c, e, f *p < 0.05; **p < 0.01 by Student’s t test. Scale bars, b, d 1 mm; f 400 μm.

We next examined whether Malat1 impacts osteogenesis in pathological conditions. In a femoral midshaft fracture model ^46^, we found that Malat1 deficiency in both *Malat1*^*−/−*^ mice and *Malat1*^*ΔOcn*^ mice significantly disturbed the bone regeneration at 21 days post-fracture, indicated by significantly decreased bone mass in the newly formed callus (Fig. 8). These results indicate that Malat1 in osteoblasts plays an important role in enhancing bone regeneration in fracture healing.

## Discussion

Malat1 stands out as one of the most abundant and evolutionarily conserved long non-coding RNAs (lncRNAs). Initially, the lack of evident defects in Malat1 knockout (KO) mice led to the assumption that Malat1 might not be essential for development and physiological processes. However, previous phenotyping studies did not encompass the examination of bone, a vital yet often overlooked organ. Despite its appearance as a hard tissue, bone undergoes continuous remodeling involving various cell types to maintain its mass and function. In this study, we investigated the function of Malat1 in bone. Our results revealed that Malat1 acts as a crucial player through the concerted actions of multiple bone cells via a new molecular network in the intricate regulation of bone homeostasis and remodeling under physiological conditions (Fig. 7). In pathological conditions, such as fracture healing, Malat1 is an important regulator that promotes bone regeneration. These findings exemplify the unprecedented key role of lncRNAs, like Malat1, in tissue homeostasis and remodeling, shedding light on their broader significance in orchestrating cellular and molecular networks for maintaining and adapting tissue homeostasis and regeneration.

During the submission of our manuscript, another group ^47^ reported that Malat1 KO mice show reduced bone mass, which is the only similar phenotype as observed in our study. However, there are many novel findings that are distinct in our study. In addition to global Malat1 KO mice, we generated both osteoblast and osteoclast specific Malat1 conditional KO mouse lines. Using the osteoblast-specific conditional KO mice (*Malat1*^*ΔOcn*^), we demonstrate that Malat1 promotes bone formation in osteoblasts. Using the myeloid osteoclast-specific mice (*Malat1*^*ΔM/ ΔM*^), we demonstrate that Malat1 does not directly affect osteoclastogenesis, but suppresses osteoclastogenesis in a non-autonomous manner in vivo by integrating crosstalk between multiple cell types, including osteoblasts, osteoclasts and chondrocytes. We further found that Malat1 binds to β-catenin and functions through the β-catenin-OPG/Jagged1 pathway in osteoblasts and chondrocytes to enhance bone formation and suppress bone resorption (Fig. 7). Moreover, Malat1 significantly promotes bone regeneration in fracture healing. Taken together, these findings, including the cellular and molecular mechanisms as well as the healing model, are novel and not reported before.

Fine-tuning in biology is essential for maintaining homeostasis ^48,49^. Bone must adapt to various environmental changes, ensure optimal function, and respond effectively and precisely to internal and external signals. In this study, we found that Malat1 is a central player in fine-tuning bone homeostasis and remodeling. Malat1 promotes osteoblastic bone formation. It also orchestrates the β-catenin pathway to activate the downstream target genes, OPG and Jagged1, which are potent osteoclastogenic inhibitors. Interestingly, Malat1 does not directly affect osteoclastogenesis in osteoclast cell lineage. The regulation of OPG and Jagged1 by Malat1 occurs in osteoblasts and chondrocytes. Malat1 alters the expression of OPG and Jagged1 in these cells, thereby impacting osteoclastogenesis in a non-autonomous manner. Therefore, Malat1 impacts not only one cell type but fine-tunes a complex cellular and molecular network in the skeletal system. In this network, OPG and Jagged1 are newly identified targets regulated by Malat1.

The Malat1-β-catenin-OPG/Jagged1 axis identified in our study represents a novel mechanism regulating the crosstalk between chondrocytes and osteoclasts. The exact location of OPG-expressing chondrocytes in cartilage remains unclear. Previous reports using traditional histochemical staining have yielded controversial results, with some indicating OPG expression in articular cartilage and others in growth plates ^50–53^. In this study, we do not focus on comparing the sources of OPG from chondrocytes in the growth plate versus articular cartilage. Future studies utilizing advanced technologies would be valuable in elucidating the spatial expression pattern of OPG in cartilage.

Although previous reports observed a link between Malat1 and β-catenin signaling pathway in cancers ^34,35^, the underlying molecular mechanisms were unclear. Specifically, it is poorly understood how Malat1 interacts with β-catenin and regulates its nuclear retention and transcriptional activity. Our studies elucidated these questions and uncovered the molecular basis of the Malat1- β-catenin pathway. With consideration that lncRNAs can utilize diverse molecular mechanisms, Malat1 may also bind to additional regulators. Future studies may involve ChIRP assays followed by mass spectrometric analysis to identify additional potential Malat1-bound targets.

While studies on lncRNAs using *in vivo* genetic approaches are increasing, many lncRNA investigations still rely heavily on *in vitro* methods, such as RNAi techniques for a knockdown in cell cultures. While a knockdown model has the advantage of facilitating high-throughput screens, emerging evidence has revealed significant non-specific or off-target effects *in vitro*. This has caused subsequent mischaracterizations of lncRNA functions and their respective mechanisms. This is particularly concerning for abundant nuclear lncRNAs, such as Malat1. Additional concerns include the transient knockdown effects *in vitro* versus the stable knockout effects *in vivo*. The definitive evidence of functionality comes from genetic knockouts, enabling the examination of *in vivo* function and minimizing the risk of off-target effects. Controversial reports exist between *in vitro* and *in vivo* studies regarding Malat1’s function in nuclear speckles and cancers ^17–21,54–57^. Recently, some *in vitro* studies suggested that Malat1 acts as a miRNA sponge ^58^. However, miRNAs are primarily located in the cytoplasm and lncRNA-miRNA interaction occurs almost exclusively in this cellular compartment. Given Malat1’s nuclear localization, it is unlikely to function as an ‘miRNA sponge’. At least, the location of the potential interaction between Malat1 and miRNAs, as well as the Malat1 copies in extra-nuclear compartments, need to be definitively verified using robust approaches. Current reports on Malat1’s functions in bone cells have similar concerns with their in vitro approaches ^55–57^. Overall, the distinct results obtained from *in vitro* knockdown systems and *in vivo* Malat1 knockout mouse models provide a clear example of the limitations of *in vitro* knockdown systems for annotating lncRNA function *in vivo*. Therefore, discussions in the lncRNA field have emphasized the robustness of approaches, and highlighted the critical need to apply genetic methods aptly to confidently characterize lncRNA functions ^17,19,54^. This is especially relevant in *in vivo* contexts such as development, metabolism, homeostasis, and tissue remodeling. The *Malat1*^*−/−*^ mice ^17^ used in this study were generated by inserting beta-galactosidase gene followed by a polyadenylation signal immediately downstream from the transcriptional start site of *Malat1*. This strategy preserves DNA regulatory elements at the *Malat1* locus, thereby avoiding confusion in result interpretation arising from the loss of these elements.

A challenge in lncRNA field is the unclear structures of most lncRNAs. The size of lncRNAs typically spans from 1kb to over 100kb. These molecules exhibit intricate secondary and tertiary structures that become increasingly dynamic and complex based on their interactions with various proteins. The structural flexibility of lncRNAs in diverse cellular contexts poses a more significant challenge in deciphering the relationship between their structure and function ^1^. For instance, in a model of estimating lncRNA structures, approximately half of Malat1 nucleotides were found to adopt various structures, including nearly 200 helices, many pseudoknots, structured tetraloops, internal loops, and intricate intramolecular long-range interactions featuring multiway junctions ^59^. To unravel these complexities, breakthrough techniques are required to understand the functions of each and all of the dynamic structures of lncRNAs.

Many skeletal diseases involve defects in bone remodeling, a process that includes multiple cell types, cellular crosstalk, and complex molecular pathways. Treatment efficacy is usually insufficient by targeting only one bone cell type. This is clearly evident in diseases such as RA and periodontitis, in which standard antiresorptive therapies, such as bisphosphonates that inhibit osteoclast activity, are not able to effectively restore bone formation ^24–27^. Therefore, there is a clinical need for new or complementary therapies that can target multiple bone cell types, such as osteoblasts and osteoclasts. These innovative therapeutic strategies go beyond targeting a single cell type, aiming instead to restore healthy bone remodeling and significantly enhance treatment efficacy. Malat1, identified in this study, emerges as a crucial fine-tuner that integrates multiple bone cells and a molecular network to maintain bone homeostasis. Thus, Malat1 and its mediated cellular and molecular mechanisms not only hold significant implications for our understanding of conditions related to bone health but also offer a potential avenue for developing more efficient treatments for bone diseases.

## Materials and Methods

### Animals

*Malat1*^*−/−* 17^ and *Malat1*^*flox/flox*^ mice ^19^ were described previously. Sex- and age-matched *Malat1*^*−/−*^ mice and their littermates WT (*Malat1*^*+/+*^) mice were used for experiments. We generated mice with osteoblast-specific deletion of *Malat1* by crossing *Malat1*^*flox/flox*^ mice with osteocalcin cre mice (The Jackson Laboratory, Stock No: 019509). Sex- and age-matched *Malat1*^*flox/flox*^ ;Ocn-Cre mice (referred to as *Malat1*^*ΔOcn*^) and their littermates *Malat1*^*flox/flox*^ mice as the controls (referred to as the *Malat1*^*f/f*^) were used for experiments. We generated mice with myeloid/macrophage-specific deletion of *Malat1* by crossing the *Malat1*^*flox/flox*^ mice with LysM Cre mice (The Jackson Laboratory, Stock No: 004781). Sex- and age-matched *Malat1*^*flox/flox*^ ;LysM-Cre mice (referred to as the *Malat1*^*ΔM /ΔM*^ mice) and their littermates with *Malat1*^*+/+*^; LysMcre (+) genotype as WT controls (hereafter referred to as Control) were used for experiments.

After sacrifice, the bones were fixed by 4% formaldehyde and subjected to μCT analysis, sectioning, TRAP staining and histological analysis. μCT analysis of femoral trabecular bones and cortical midshaft was conducted to evaluate bone volume and 3D bone architecture using a Scanco μCT-35 scanner (SCANCO Medical) according to the manufacturer’s instructions and the American Society of Bone and Mineral Research (ASBMR) guidelines ^60^.

For dynamic histomorphometric measures of bone formation ^61^, calcein (25 mg/kg, Sigma) was injected into mice intraperitoneally at 5 and 2 days before sacrifice to obtain double labeling of newly formed bones. The non-decalcified tibia bones were embedded in methyl methacrylate. 5 mm thick sections were sliced using a microtome (Leica RM2255, Leica Microsystems, Germany). For static histomorphometric measures of osteoblast parameters, non-decalcified sections of the tibiae were stained using toluidine blue or Masson-Goldner staining kit (MilliporeSigma). The Osteomeasure software was used for bone histomorphometry using standard procedures according to the program’s instruction.

All mice were housed in a 12h:12h light/dark cycle with food and water ad libitum. All animal procedures were performed according to the approved protocol (2016–0001 and 0004) by the Institutional Animal Care and Use Committee (IACUC) of Hospital for Special Surgery and Weill Cornell Medical College.

### Bone fracture model

Bone fracture was performed as described previously with slight modifications ^46^. 13-week-old mice were anesthetized with 2.5% isoflurane via inhalation. The mice received meloxicam (subcutaneous injection, 2 mg/kg) and buprenorphine (subcutaneous injection, 0.5mg/kg) as analgesia prior to surgery. The surgical site was shaved and sterilized using iodine and 70% ethanol. An incision was made over the anterolateral femur. 0.1 ml bupivacaine (5mg/ml) was injected into the tissue adjacent to the incision line. A 25-gauge needle was inserted into the femoral canal through the patellar groove. The mid-diaphysis of the femur was transected using dental drill with a burr (19007–05, Fine science tools). The needle was then trimmed from the distal end to prevent it from projecting into the femoral joint space. The left part of the needle remained in femur to stabilize the fracture. The muscle was repositioned over the injury site and stitched using absorbable sutures. The skin was closed with wound clips, which were removed 2 weeks post-surgery. Animals were administered 0.5mg/kg buprenorphine every 12 hours and 2.0 mg/kg meloxicam every 24 hours subcutaneously for analgesia up to 3 days after the surgery. Mice were sacrificed 21 days post-fracture. Hematoxylin and eosin (HE) staining of the bone samples were performed. BV/TV of the callus was analyzed using μCT and Osteomeasure on the tissue slices. All surgical procedures were performed according to the approved protocol (2016–0001/4) by the Institutional Animal Care and Use Committee (IACUC) of Hospital for Special Surgery and Weill Cornell Medical College.

### Reagents

Murine M-CSF (Cat# 315–02), recombinant human sRANK Ligand (Cat# 310–01), recombinant human OPG (Cat# 450–14) and murine Wnt3a (Cat# 315–20) were purchased from PeproTech. Leukocyte Acid Phosphatase (TRAP) Kit (Cat# 387A) and Mouse Osteoprotegerin ELISA Kit (Cat# RAB0493) were obtained from MilliporeSigma. The plasmid of M50 Super 8x TOPFlash (Cat# 12456) was purchased from Addgene. pRL-Tk control plasmid (Cat# E2241) was purchased from Promega. Fetal Bovine Serum (FBS, Cat #S11550) was obtained from Atlanta Biologicals. β-glycerophosphate disodium salt hydrate (Cat# G9422), L-ascorbic acid (Cat# A4544), prostaglandin E2 (PGE2) (Cat# P0409) and 1α,25-Dihydroxyvitamin D3 (VitD3) (Cat# D1530) were obtained from MilliporeSigma.

### Cell culture

For osteoclastogenesis, bone marrow cells were obtained from age and sex-matched WT and *Malat1*^*−/−*^ littermates and cultured in αMEM supplemented with 10% FBS and 2.4 mM glutamine (25030081, Thermo Fisher Scientific) and 1% Penicillin–Streptomycin along with CMG14–12 supernatant ^62^, serving as the condition medium (CM) which contained a concentration equivalent to 20 ng/ml of rM-CSF and was utilized as the M-CSF source. After 3-day culture, the cells were washed in 1x PBS and the attached cells were scraped, and seeded into plates at a density of 4.5 × 10^4^/cm^2^ with CM. Next day, the cells were induced for osteoclastogenesis with 40 ng/ml RANKL and CM for the indicated days shown in figures. Multi-nucleated osteoclasts were stained using Leukocyte Acid Phosphatase (TRAP) Kit (Cat# 387A, MilliporeSigma) according to the manufacturer’s instructions.

Primary osteoblastic cells were isolated from the calvaria of new-born (0–3d) mice by enzymatic digestion in 10% FBS αMEM with 0.1% collagenase (LS004177, Worthington) and 0.2% dispase (17105041, Thermo Fisher Scientific) as described ^61^. The cells were used immediately or cultured to expand for 6 days for experiments indicated in relevant figures.

For the co-cultures of primary osteoblasts and bone marrow cells in transwell plates, primary calvarial osteoblasts isolated from WT and *Malat1*^*−/−*^ newborn mice were seeded in the upper chamber (2 × 10^4^ cells/96-well) in αMEM medium with 10% FBS. After primary osteoblasts reached 60–70% confluence, bone marrow cells harvested from the femur and tibia of 6-week-old mice were plated to the bottom chamber (1 × 10^6^ cells/24-well), together with 1 μM Prostaglandin E2 (PGE2) (P0409, MilliporeSigma) and 10nM 1α,25-Dihydroxyvitamin D3 (VitD3) (D1530, MilliporeSigma). Culture media were exchanged every two days for 12 days. TRAP staining was performed and multinucleated osteoclasts were counted.

For the direct co-cultures of primary osteoblasts and bone marrow cells, primary osteoblasts isolated from WT and *Malat1*^*−/−*^ newborn mice were seeded in 48-well plates at a density of 2 × 10^4^ cells/well in αMEM supplemented with 10% FBS. After osteoblasts reached 60–70% confluence, murine bone marrow cells isolated from the femur and tibia of 6-week-old mice were plated at a density of 2 × 10^5^ cells/well on the top the osteoblasts in the presence of 1 μM PGE2 and 10nM VitD3. Culture media were exchanged every two days. On the sixth day, TRAP staining was performed and multinucleated osteoclasts were counted.

Preparation and culture of primary mouse chondrocytes were performed as previously described with slight modification ^63^. In brief, femoral condyles and tibial plateau were carefully dissected from 5-day-old WT and *Malat1*^*−/−*^ mice. Soft tissues were removed under a microscope. After washing with PBS for two times, the femoral condyles and tibial plateau were first digested in digestion buffer (αMEM containing 10% FBS, 1 mg/ml collagenase II, and 2 mg/ml Dispase I) in 37 °C incubator for 45 min. Then, the femoral condyles and tibial plateau were transferred to fresh digestion buffer and digested overnight in 37 °C incubator. The next day, the isolated chondrocytes were filtered through a 70 μm cell strainer and spun down at 1600 rpm for 5 min. The chondrocytes were directly used for RNA extraction, seeded for Alican blue staining (A5268, MilliporeSigma) in 24-well plates, or for immunofluorescence staining of Aggrecan (A8536, ABclonal) in 96-well plates.

MC3T3-E1 osteoblasts were purchased from ATCC and cultured in αMEM with 10% FBS and 1% Penicillin–Streptomycin (15140122, Thermo Fisher Scientific).

### Production of Wnt3a conditioned medium

The L Wnt-3A (CRL-2647, ATCC) cells and the control L cell line (CRL-2648, ATCC) were generously gifted by Dr. Joe Zhou (Weill Cornell Medicine). We followed ATCC handling information to maintain and culture the cells. Briefly, the cells were maintained in DMEM medium supplemented with 10% FBS, 1% Penicillin–Streptomycin and 0.4 mg/ml G-418. To produce the Wnt3a conditioned medium (Wnt3a CM) and control medium (L CM), the cultured cells (80–90% confluence) from one 10cm dish were split at a 1:10 ratio and seeded into 10 cm tissue culture dishes. After an initial 4-day culture (approximately to confluency) in DMEM medium containing 10% FBS and 1% Penicillin–Streptomycin, the first batch of medium was harvested. Subsequently, 10 ml of fresh medium was added, and the cells were cultured for another 3 days to collect the second batch of conditioned medium. The two batches of conditioned media were combined at a 1:1 ratio, filtered using a 0.22 μm filter, and stored at −80°C. Wnt3a activity was confirmed by nuclear translocation of β-catenin.

### Immunofluorescence staining

Primary osteoblasts or chondrocytes were seeded into 96-well plates at a density of 1 × 10^4^ cells/well. Primary osteoblasts were serum starved for 16 h, followed by treatment with 50% L- or 50% Wnt3a CM for 1h. After washing with PBS, the cells were fixed with 4% paraformaldehyde in PBS for 20 min at room temperature, permeabilized with 0.5% Triton X-100 in PBS for 15 min at room temperature, and blocked for 1 h with 1% BSA in PBS (blocking buffer). The cells were then incubated with primary antibodies that were diluted in blocking buffer for overnight at 4 °C. After washing three times with PBS, the cells were incubated with secondary antibodies for 1 h at room temperature. After three times of washing with PBS, the cells were mounted with the ProLong^™^ Gold Antifade Mountant with DAPI (P36941, ThermoFisher Scientific). A Zeiss microscope was used to take images. Primary antibodies include anti-β-catenin antibody (8480s, Cell Signaling Technology, 1:100) and anti-Aggrecan (A8536, ABclonal,1:100) in this study. The goat anti-Rabbit Alexa Fluor^™^ 488 (A-11008, ThermoFisher Scientific, diluted at 1:500 with blocking buffer) was used as the secondary antibody for these primary antibodies.

### Immunoblot analysis

Total cellular extracts were obtained using lysis buffer containing 150 mM Tris-HCl (pH 6.8), 6% SDS, 30% glycerol, and 0.03% Bromophenol Blue, with 10% 2-Mercaptoethanol added immediately before harvesting cells. Cell lysates were fractionated on 7.5% SDS-PAGE, transferred to Immobilon-P membranes (0.45 μm, Millipore), and incubated with specific antibodies. Western Lightning Plus-ECL (PerkinElmer) was used for detection. β-catenin antibody (9562, 1:1000) and Jag1 antibody (70109, 1:1000) were obtained from Cell Signaling Technology. Nfatc1 antibody (556602, 1:1000) was obtained from BD Biosciences; Blimp1 (sc-47732, 1:1000), c-Fos (sc-52, 1:1000), OPG/Osteoprotegerin (sc-390518, 1:1000) and p38α (sc-535, 1:3000) antibodies were purchased from Santa Cruz Biotechnology.

### Cytoplasmic and nuclear extraction

The cultured primary osteoblastic cells were subjected to serum starvation in αMEM with 2% FBS for 16h. Subsequently, the cells were treated with either 50% L- or 50% Wnt3a conditioned medium (CM) for 1 hour. The cells were collected and lysed with Buffer A (10mM Hepes PH7.9, 1.5mM MgCl_2_, 10mM KCl) supplemented with fresh protease inhibitor cocktail (11836170001, MilliporeSigma). After incubation on ice for 15 min, 0.2 % NP-40 was added. The mixture was vortexed and incubated on ice for 2 min. The cellular lysate was then centrifuged at 10,000 g for 3 minutes at 4°C. The supernatant was used as cytoplasmic fraction. The nuclei pellets were washed two times using 1 ml of Buffer A. After the second wash, the buffer was completely removed. The nuclei pellet was then lysed using Buffer C (20mM Hepes pH7.9, 1.5mM MgCl_2_, 420mM NaCl, 0.2mM EDTA, 25% Glycerol) with fresh protease inhibitor cocktail for 30 minutes on ice, vortexing every 5 minutes. The nuclear lysate was centrifuged at 12,000 × g at 4°C for 15 minutes, and the supernatant was collected as the nuclear extract. GAPDH (sc-25778) and TBP1(sc-204) were purchased from Santa Cruz Biotechnology and used as the cytoplasmic and nuclear markers, respectively. β-catenin antibody (9562, 1:1000) was obtained from Cell Signaling Technology.

### Reverse transcription and real-time PCR

DNA-free RNAs were isolated from cells with the RNeasy MiniKit (74104, Qiagen) with DNase treatment, and total RNA was reverse-transcribed with random hexamers using the RevertAid RT Kit (K1691, Thermo Fisher Scientific) according to the manufacturer’s instructions. Real-time PCR was done in triplicate with the QuantStudio 5 Real-time PCR system (A28138, Applied Biosystems) and Fast SYBR^®^ Green Master Mix (4385612, Thermo Fisher Scientific) with 500 nM primers. mRNA amounts were normalized relative to glyceraldehyde-3-phosphate dehydrogenase (GAPDH) mRNA. The mouse primers for real-time PCR were as follows: *Gapdh*: 5’-ATCAAGAAGGTGGTGAAGCA-3’ and 5’-AGACAACCTGGTCCTCAGTGT-3’; *Tnfrsf11b*: 5’-CGGAAACAGAGAAGCCACGCAA-3′ and 5’-CTGTCCACCAAAACACTCAGCC-3′; *Tnfsf11*: 5’-CAGCATCGCTCTGTTCCTGTA-3′ and 5’-CTGCGTTTTCATGGAGTCTCA-3′; *Axin2*: 5’-ATGCAAAAGCCACCCAAAGG-3′ and 5’-TGCATTCCGTTTTGGCAAGG-3′; *Ccnd1*: 5’-GCGTACCCTGACACCAATCTC-3′ and 5’-CTCCTCTTCGCACTTCTGCTC-3′; *Lef1*: 5’-TGTTTATCCCATCACGGGTGG-3′ and 5’-CATGGAAGTGTCGCCTGACAG-3′; c-myc: 5’-CAGCGACTCTGAAGAAGAGCA-3′ and 5’-TTGTGCTGGTGAGTGGAGAC-3′; *Jag1*: 5’-TGCCTGCCGAACCCCTGTCATAAT-3’ and 5’-CCGATACCAGTTGTCTCCGTCCAC-3’; *Tcf7*: AACTGGCCCGCAAGGAAAG and CTCCGGGTAAGTACCGAATGC;*Nfatc1*: 5′-CCCGTCACATTCTGGTCCAT-3′ and 5′-CAAGTAACCGTGTAGCTCCACAA-3′; *Acp5*: 5′-ACGGCTACTTGCGGTTTC-3′ and 5′-TCCTTGGGAGGCTGGTC-3′; *Ctsk:* 5′-AAGATATTGGTGGCTTTGG-3′ and 5′-ATCGCTGCGTCCCTCT-3′; *Dc-stamp*: 5′-TTTGCCGCTGTGGACTATCTGC-3′ and 5′-AGACGTGGTTTAGGAATGCAGCTC-3′; *Blimp1*: 5’-TTCTTGTGTGGTATTGTCGGGACTT-3′ and 5’-TTGGGGACACTCTTTGGGTAGAGTT-3′; *Atp6v0d2*: 5’-GAAGCTGTCAACATTGCAGA-3′ and 5’-TCACCGTGATCCTTGCAGAAT-3′; *Malat1*: 5’-AGCAGGCATTGTGGAGAGGA-3′ and 5’-ATGTTGCCGACCTCAAGGAA-3′; *Col2a1*: CGATCACAGAAGACCTCCCG and GCGGTTGGAAAGTGTTTGGG; *Sox9:* AAGCTCTGGAGGCTGCTGAACGAG and CGGCCTCCGCTTGTCCGTTCT; *Acan*: GGTCACTGTTACCGCCACTT and CCCCTTCGATAGTCCTGTCA.

### RNA immunoprecipitation (RIP) assay

Thirty million MC3T3-E1 cells were collected, centrifuged, and washed with PBS. The cells were then spun down at 800×g for 4 min at room temperature. The cell pellet was resuspended in 1% formaldehyde in PBS (28906, Thermo Fisher Scientific) and crosslinked for 10 min at room temperature on an end-to-end rotator. 1.25M glycine at 1/10 volume of 1% formaldehyde solution was used to quench the cross-linking reaction at room temperature for 5 min. The cells were spun down at 2000 × g for 5 min and the pellet was washed with chilled PBS once, followed by centrifugation at 2000 × g for 5 min. The cell pellets were resuspended in 1.1 ml immunoprecipitation (IP) lysis buffer (50 mM HEPES at pH 7.5, 0.4 M NaCl, 1 mM EDTA, 1 mM DTT, 0.5% Triton X-100, 10% glycerol) containing 1 mM PMSF (78830, MilliporeSigma), protease inhibitor cocktail, and RNase inhibitor (100 U/ml Superase-in, AM2694, Thermo Fisher Scientific). The cell lysate was then sonicated using the Bioruptor^®^ Pico sonication device (Diagenode, NJ, USA) until the liquid became clear (sonication cycle: 30 sec ON, 30 sec OFF). The lysates were centrifuged for 10 min at 14,000 × g at room temperature. 50 μl of the supernatant was used as the input control. The remaining supernatant was precleared using protein A/G agarose (sc-2003, Santa Cruz Biotechnology). The precleared cell lysate was split into two tubes evenly and incubated with 5μg of β-catenin antibody (9562, Cell signaling technology) or normal rabbit IgG (2729S, Cell signaling technology) at 4 °C overnight with rotation. 30 μl of washed protein A/G beads were then added into each sample and incubated at 4 °C for 1 h with rotation. The beads were washed with 900 μl of IP lysis buffer for 3min/each time for 5 times and collected by centrifugation for 3 min at 400 × g at room temperature. After the last wash, 100μl of RIP buffer (50 mM HEPES at pH 7.5, 0.1 M NaCl, 5 mM EDTA, 10 mM DTT, 0.5% Triton X-100, 10% glycerol, 1% SDS) with 1μl RNase inhibitor was added to each sample. 50 μl of RIP buffer was added to the input control sample. All samples were incubated at 70 °C for 1 h to reverse crosslinking. 100 μl of supernatant were then collected by spinning down the beads at 400 × g for 1 min at room temperature. The supernatant was used for RNA extraction using the RNeasy Mini Kit (Qiagen) with DNase I treatment. RNA was eluted using 12ul RNase-free H2O and reversed to complementary DNA using One-step cDNA synthesis kit (Thermo, Revert Aid RT kit, k1691). The qPCR was then performed using Malat1 primers.

### Chromatin Isolation by RNA Purification (ChIRP) assay

Chromatin Isolation by RNA Purification (ChIRP) was performed as described previously with slight modifications ^64^. We designed and synthesized an antisense oligonucleotide probe of murine *Malat1.* The design was based on the high sequence homology to a human *MALAT1* probe’s sequence ^65^ (Supplementary Fig. 3). The murine *Malat1* probe’s sequence is 5’-GTCTTTCCTGCCTTAAAGTTAATTTCG/iSp18//3’-BiotinTEG (INTEGRATED DNA Technologies). We also synthesized a GFP probe (sequence: 5’ TATCACCTTCAAACTTGACTTC/ iSp18–3’-Biotin TEG) as the negative control. 30 million MC3T3-E1 cells were collected, washed in PBS and centrifuged at 800×g for 4 min at room temperature. The cell pellet was resuspended in 4% formaldehyde in PBS and crosslinked for 30 min at room temperature on an end-to-end rotator. 1.25M glycine at 1/10 volume of 4% formaldehyde solution was used to quench the crosslinking reaction at room temperature for 5 min. After washing with chilled PBS once, the cells were resuspended in 1 ml lysis buffer (50 mM Tris-Cl pH 7.0, 10 mM EDTA, 1% SDS) supplement with 1 mM PMSF, protease inhibitors, and RNase inhibitor. The cell lysate was sonicated using the Bioruptor until the lysate became clear (sonication cycle: 30 sec ON, 30 sec OFF). The lysate was centrifuged for 10 min at 14,000 × g at 4 °C. The sonicated lysate was then precleared with magnetic streptavidin beads (65001, Thermo Fisher Scientific) at 37 °C for 30 min with slow rotation. 2% volume of precleared lysate was saved for the input. The remaining lysate was split into two new tubes evenly and incubated with 100pmol of the Malat1 probe or the negative control probe in the hybridization buffer (750 mM NaCl, 1% SDS, 50 mM Tris-Cl pH 7.0, 1 mM EDTA, 15% formamide containing 1 mM PMSF, protease inhibitor cocktail (11836170001, MilliporeSigma), and RNase inhibitor (100 U/ml Superase-in, AM2694, Thermo Fisher Scientific)) at 37 °C for overnight with slow rotation. Magnetic streptavidin beads were then added to the samples and incubated at 37 °C for 30 min with slow rotation. DynaMag-15 magnetic strip was used to separate beads from the liquids. After 5 times of washing using the wash buffer (2x NaCl and Sodium citrate (SSC, 15557044, Thermo Fisher Scientific), 0.5% SDS) with PMSF and proteinase inhibitor cocktail, the beads were isolated. The bound proteins were eluted by boiling the beads in 30ul of 3x blue juice buffer (150 mM Tris-HCl (pH 6.8), 6% SDS, 30% glycerol, and 0.03% Bromophenol Blue, with fresh 10% 2-Mercaptoethanol) and subjected to immunoblot analysis.

### Luciferase reporter assay

3.5×10^4^ of primary calvarial osteoblasts were plated in 48-well plates. Next day, 480 ng of the M50 Super 8x TOPFlash plasmid (12456, Addgene) and 20ng of pRL-Tk control plasmid (E2241, Promega) were transfected per well using Lipofectamine 3000 (L3000001, Invitrogen) according to the manufacturer’s instructions. After 24h transfection, the culture medium was replaced with fresh αMEM medium supplemented with 10%FBS. 48h post transfection, the cells were serum starved for 1h and then treated with 20% L- or 20% Wnt3a CM for 16h. Firefly and Renilla luciferase activities were measured using a Dual-Luciferase Reporter Assay system (E1910, Promega) on a Gen5 Microplate Reader (BioTek) according to the manufacturer’s instructions. Firefly luciferase activity was normalized to Renilla luciferase activity.

### Single cell RNAseq (scRNAseq) Analysis

Single cell RNAseq datasets for bone (GSM3674239, GSM3674240, GSM3674241, GSM3674242) and bone marrow (GSM3674243, GSM3674244, GSM3674246) were downloaded from GSE128423 ^42^. Seurat package ^66^ was applied for downstream analysis. Briefly, genes expressed in fewer than 3 cells and cells with less than 500 genes were filtered out. Cells with over 10% mitochondrial reads and exceeding 6,000 nFeature_RNA were excluded. After NormalizeData, the top 5000 variable genes were selected based on dispersion method using FindVariableGenes function of Seurat package. Subsequently, data scaling was performed using the ScaleData function. All datasets were integrated based on the identified anchors. The first 30 principal components for both UMAP (Uniform Manifold Approximation and Projection) and the subsequent application of a graph-based clustering approach were used, with resolution at 0.1. The Clustree function was executed to understand how the structure of clusters changes across different resolutions. The FindAllMarkers function was utilized with parameters set to prioritizing positive markers expressed in at least 10% of cells within a cluster and exhibiting a log-fold change threshold of 0.25. The following gene markers were also included for cluster annotation: *Acan, Col2a1 and Sox9* for chondrocytes ^42^; *Acta2, Myh11* and *Mcam* for pericytes ^42^; *Adipoq, Lepr, Cxcl12, Cebpa, Kitl* and *Lpl* for Adipoq-lineage progenitors ^67^; *Prrx1, Col1a1, Ibsp* and *Bglap* for osteoblast lineage ^67^; *Pecam1* and *Cdh5* for endothelial cells ^67^; *Cd19* for B cells ^67^; *S100a8* for neutrophils ^67^; *Mpz, Mbp* and *Plp1* for Schwann cells ^68^; *Gypa, Alas2, Snca, Hbb-bs, Hbb-bt, Car1, Car2, Klf1, Gata1* and *Gata2* for Erythroid cells ^69–72^; *Pf4, Itga2b* and *Fli1* for Megakaryocytes ^72^; *Cd68, Lyz2, Ly6c2* and *Sell* for Monocyte-Macrophage lineage^67,73^. We utilized Seurat’s FeaturePlot to visualize gene expression in individual cells. DotPlot was employed to illustrate the percentage of cells in a cluster expressing a specific gene and to visualize the average scaled expression level of each gene. The distribution of normalized gene expression levels across all cells in each cluster was visualized using VlnPlot function in Seurat. R version 4.3.2 and Seurat 5.0.1 were used in the study.

### Bone marrow supernatant collection

Bone marrow from 12-week-old WT and *Malat1*^*−/−*^ littermate mice was harvested from the femur and tibia (with both ends cut open) by flash centrifugation. The cell pellets were resuspended in 0.2 ml of chilled PBS containing protease inhibitor cocktail (11836170001, MilliporeSigma) and incubated on ice for 30 min. The suspension was centrifuged at 1,500 rpm for 15 min at 4°C and the supernatant was collected for immunoblot analysis.

### ELISA

Mouse serum OPG, P1NP and TRAP were measured using Mouse Osteoprotegerin ELISA Kit (MilliporeSigma), P1NP and TRAP ELISA kits (MyBioSource), respectively, according to the manufacturer’s instruction.

### Statistical analysis

Statistical analysis was performed using Graphpad Prism^®^ software. Two-tailed Student’s t test was applied when there were only two groups of samples. In the case of more than two groups of samples, one-way ANOVA will be used with one condition, and two-way ANOVA was used with more than one condition. ANOVA analysis was followed by post hoc Bonferroni’s correction for multiple comparisons. p < 0.05 was taken as statistically significant. Data are presented as the mean ± SD as indicated in the figure legends.

## Supplementary Material

Supplement 1

## Data Availability

All data supporting the findings of this study are available within the paper, its Supplementary Information, and source data file. The sequence dataset from GSE128423 ^42^ was reanalyzed.

## References

[1] Mattick J.S. Long non-coding RNAs: definitions, functions, challenges and recommendations. Nat Rev Mol Cell Biol 24, 430–447 (2023). 36596869 10.1038/s41580-022-00566-8 PMC10213152

[2] Batista P.J. & Chang H.Y. Long noncoding RNAs: cellular address codes in development and disease. Cell 152, 1298–307 (2013). 23498938 10.1016/j.cell.2013.02.012 PMC3651923

[3] Quinn J.J. & Chang H.Y. Unique features of long non-coding RNA biogenesis and function. Nat Rev Genet 17, 47–62 (2016). 26666209 10.1038/nrg.2015.10

[4] Smith M.A. & Mattick J.S. Structural and Functional Annotation of Long Noncoding RNAs. Methods Mol Biol 1526, 65–85 (2017). 27896736 10.1007/978-1-4939-6613-4_4

[5] Rinn J.L. & Chang H.Y. Genome regulation by long noncoding RNAs. Annu Rev Biochem 81, 145–66 (2012). 22663078 10.1146/annurev-biochem-051410-092902 PMC3858397

[6] Cech T.R. & Steitz J.A. The noncoding RNA revolution-trashing old rules to forge new ones. Cell 157, 77–94 (2014). 24679528 10.1016/j.cell.2014.03.008

[7] Schmitt A.M. & Chang H.Y. Long Noncoding RNAs: At the Intersection of Cancer and Chromatin Biology. Cold Spring Harb Perspect Med 7(2017). 10.1101/cshperspect.a026492 PMC5495049 28193769

[8] Modarresi F. Inhibition of natural antisense transcripts in vivo results in gene-specific transcriptional upregulation. Nat Biotechnol 30, 453–9 (2012). 22446693 10.1038/nbt.2158 PMC4144683

[9] Shappell S.B. Clinical utility of prostate carcinoma molecular diagnostic tests. Rev Urol 10, 44–69 (2008). 18470278 PMC2312347

[10] Tham Y.K., Bernardo B.C., Ooi J.Y., Weeks K.L. & McMullen J.R. Pathophysiology of cardiac hypertrophy and heart failure: signaling pathways and novel therapeutic targets. Arch Toxicol 89, 1401–38 (2015). 25708889 10.1007/s00204-015-1477-x

[11] Wheeler T.M. Targeting nuclear RNA for in vivo correction of myotonic dystrophy. Nature 488, 111–5 (2012). 22859208 10.1038/nature11362 PMC4221572

[12] Wu G.C. Emerging role of long noncoding RNAs in autoimmune diseases. Autoimmun Rev 14, 798–805 (2015). 25989481 10.1016/j.autrev.2015.05.004

[13] Yang L., Froberg J.E. & Lee J.T. Long noncoding RNAs: fresh perspectives into the RNA world. Trends Biochem Sci 39, 35–43 (2014). 24290031 10.1016/j.tibs.2013.10.002 PMC3904784

[14] Gutschner T. The noncoding RNA MALAT1 is a critical regulator of the metastasis phenotype of lung cancer cells. Cancer Res 73, 1180–9 (2013). 23243023 10.1158/0008-5472.CAN-12-2850 PMC3589741

[15] Yoshimoto R., Mayeda A., Yoshida M. & Nakagawa S. MALAT1 long non-coding RNA in cancer. Biochim Biophys Acta 1859, 192–9 (2016). 26434412 10.1016/j.bbagrm.2015.09.012

[16] Kwok Z.H., Roche V., Chew X.H., Fadieieva A. & Tay Y. A non-canonical tumor suppressive role for the long non-coding RNA MALAT1 in colon and breast cancers. Int J Cancer 143, 668–678 (2018). 29574704 10.1002/ijc.31386

[17] Nakagawa S. Malat1 is not an essential component of nuclear speckles in mice. RNA 18, 1487–99 (2012). 22718948 10.1261/rna.033217.112 PMC3404370

[18] Zhang B. The lncRNA Malat1 is dispensable for mouse development but its transcription plays a cis-regulatory role in the adult. Cell Rep 2, 111–23 (2012). 22840402 10.1016/j.celrep.2012.06.003 PMC3408587

[19] Eissmann M. Loss of the abundant nuclear non-coding RNA MALAT1 is compatible with life and development. RNA Biol 9, 1076–87 (2012). 22858678 10.4161/rna.21089 PMC3551862

[20] Tripathi V. The nuclear-retained noncoding RNA MALAT1 regulates alternative splicing by modulating SR splicing factor phosphorylation. Mol Cell 39, 925–38 (2010). 20797886 10.1016/j.molcel.2010.08.011 PMC4158944

[21] Ip J.Y. & Nakagawa S. Long non-coding RNAs in nuclear bodies. Dev Growth Differ 54, 44–54 (2012). 22070123 10.1111/j.1440-169X.2011.01303.x

[22] Sims N.A. & Martin T.J. Coupling the activities of bone formation and resorption: a multitude of signals within the basic multicellular unit. Bonekey Rep 3, 481 (2014). 24466412 10.1038/bonekey.2013.215 PMC3899560

[23] Raggatt L.J. & Partridge N.C. Cellular and molecular mechanisms of bone remodeling. J Biol Chem 285, 25103–8 (2010). 20501658 10.1074/jbc.R109.041087 PMC2919071

[24] Goldring S.R. Bone remodelling in inflammatory arthritis. Ann Rheum Dis 72 Suppl 2, ii52–5 (2013). 23253928 10.1136/annrheumdis-2012-202199

[25] Schett G. & Gravallese E. Bone erosion in rheumatoid arthritis: mechanisms, diagnosis and treatment. Nat Rev Rheumatol 8, 656–64 (2012). 23007741 10.1038/nrrheum.2012.153 PMC4096779

[26] Schett G. & Sieper J. Inflammation and repair mechanisms. Clin Exp Rheumatol 27, S33–5 (2009). 19822043

[27] Walsh N.C. Osteoblast function is compromised at sites of focal bone erosion in inflammatory arthritis. J Bone Miner Res 24, 1572–85 (2009). 19338457 10.1359/jbmr.090320

[28] Zhong Z., Ethen N.J. & Williams B.O. WNT signaling in bone development and homeostasis. Wiley Interdiscip Rev Dev Biol 3, 489–500 (2014). 25270716 10.1002/wdev.159 PMC4199871

[29] Chen J. & Long F. beta-catenin promotes bone formation and suppresses bone resorption in postnatal growing mice. J Bone Miner Res 28, 1160–9 (2013). 23188722 10.1002/jbmr.1834 PMC3631304

[30] Holmen S.L. Essential role of beta-catenin in postnatal bone acquisition. J Biol Chem 280, 21162–8 (2005). 15802266 10.1074/jbc.M501900200

[31] Kramer I. Osteocyte Wnt/beta-catenin signaling is required for normal bone homeostasis. Mol Cell Biol 30, 3071–85 (2010). 20404086 10.1128/MCB.01428-09 PMC2876685

[32] Wang Y. Wnt and the Wnt signaling pathway in bone development and disease. Front Biosci (Landmark Ed) 19, 379–407 (2014). 24389191 10.2741/4214 PMC4000238

[33] Monroe D.G., McGee-Lawrence M.E., Oursler M.J. & Westendorf J.J. Update on Wnt signaling in bone cell biology and bone disease. Gene 492, 1–18 (2012). 22079544 10.1016/j.gene.2011.10.044 PMC3392173

[34] Li H. LncRNA MALAT1 modulates ox-LDL induced EndMT through the Wnt/beta-catenin signaling pathway. Lipids Health Dis 18, 62 (2019). 30871555 10.1186/s12944-019-1006-7 PMC6417088

[35] Zhang Z.C. Targeting LncRNA-MALAT1 suppresses the progression of osteosarcoma by altering the expression and localization of beta-catenin. J Cancer 9, 71–80 (2018). 29290771 10.7150/jca.22113 PMC5743713

[36] Zhao B. Interferon regulatory factor-8 regulates bone metabolism by suppressing osteoclastogenesis. Nat Med 15, 1066–71 (2009). 19718038 10.1038/nm.2007 PMC2755267

[37] Boyce B.F., Xing L. & Chen D. Osteoprotegerin, the bone protector, is a surprising target for beta-catenin signaling. Cell Metab 2, 344–5 (2005). 16330319 10.1016/j.cmet.2005.11.011 PMC2647984

[38] Glass D.A. 2nd, Canonical Wnt signaling in differentiated osteoblasts controls osteoclast differentiation. Dev Cell 8, 751–64 (2005). 15866165 10.1016/j.devcel.2005.02.017

[39] Simonet W.S. Osteoprotegerin: a novel secreted protein involved in the regulation of bone density. Cell 89, 309–19 (1997). 9108485 10.1016/s0092-8674(00)80209-3

[40] Boyce B.F. & Xing L. Functions of RANKL/RANK/OPG in bone modeling and remodeling. Arch Biochem Biophys 473, 139–46 (2008). 18395508 10.1016/j.abb.2008.03.018 PMC2413418

[41] Tsukasaki M. OPG Production Matters Where It Happened. Cell Rep 32, 108124 (2020). 32905763 10.1016/j.celrep.2020.108124

[42] Baryawno N. A Cellular Taxonomy of the Bone Marrow Stroma in Homeostasis and Leukemia. Cell 177, 1915–1932 e16 (2019). 31130381 10.1016/j.cell.2019.04.040 PMC6570562

[43] Kopan R. & Ilagan M.X. The canonical Notch signaling pathway: unfolding the activation mechanism. Cell 137, 216–33 (2009). 19379690 10.1016/j.cell.2009.03.045 PMC2827930

[44] Bai S. NOTCH1 regulates osteoclastogenesis directly in osteoclast precursors and indirectly via osteoblast lineage cells. J Biol Chem 283, 6509–18 (2008). 18156632 10.1074/jbc.M707000200

[45] Zhao B. TNF and Bone Remodeling. Curr Osteoporos Rep 15, 126–134 (2017). 28477234 10.1007/s11914-017-0358-z PMC6408950

[46] Xu R. Targeting skeletal endothelium to ameliorate bone loss. Nat Med 24, 823–833 (2018). 29785024 10.1038/s41591-018-0020-z PMC5992080

[47] Zhao Y. Long noncoding RNA Malat1 protects against osteoporosis and bone metastasis. Nat Commun 15, 2384 (2024). 38493144 10.1038/s41467-024-46602-3 PMC10944492

[48] Lin J.X. & Leonard W.J. Fine-Tuning Cytokine Signals. Annu Rev Immunol 37, 295–324 (2019). 30649989 10.1146/annurev-immunol-042718-041447 PMC10822674

[49] Summers deLuca L. & Gommerman J.L. Fine-tuning of dendritic cell biology by the TNF superfamily. Nat Rev Immunol 12, 339–51 (2012). 22487654 10.1038/nri3193

[50] Park D.R. Osteoclast-associated receptor blockade prevents articular cartilage destruction via chondrocyte apoptosis regulation. Nat Commun 11, 4343 (2020). 32859940 10.1038/s41467-020-18208-y PMC7455568

[51] Chen D., Liu Y., Liu Z. & Wang P. OPG is Required for the Postnatal Maintenance of Condylar Cartilage. Calcif Tissue Int 104, 461–474 (2019). 30623241 10.1007/s00223-018-00510-z

[52] Kishimoto K., Kitazawa R., Kurosaka M., Maeda S. & Kitazawa S. Expression profile of genes related to osteoclastogenesis in mouse growth plate and articular cartilage. Histochem Cell Biol 125, 593–602 (2006). 16283360 10.1007/s00418-005-0103-z

[53] Silvestrini G. Detection of osteoprotegerin (OPG) and its ligand (RANKL) mRNA and protein in femur and tibia of the rat. J Mol Histol 36, 59–67 (2005). 15704000 10.1007/s10735-004-3839-1

[54] Kim J. Long noncoding RNA MALAT1 suppresses breast cancer metastasis. Nat Genet 50, 1705–1715 (2018). 30349115 10.1038/s41588-018-0252-3 PMC6265076

[55] Cui Y. EPC-derived exosomes promote osteoclastogenesis through LncRNA-MALAT1. J Cell Mol Med 23, 3843–3854 (2019). 31025509 10.1111/jcmm.14228 PMC6533478

[56] Yang X., Yang J., Lei P. & Wen T. LncRNA MALAT1 shuttled by bone marrow-derived mesenchymal stem cells-secreted exosomes alleviates osteoporosis through mediating microRNA-34c/SATB2 axis. Aging (Albany NY) 11, 8777–8791 (2019). 31659145 10.18632/aging.102264 PMC6834402

[57] Yi J., Liu D. & Xiao J. LncRNA MALAT1 sponges miR-30 to promote osteoblast differentiation of adipose-derived mesenchymal stem cells by promotion of Runx2 expression. Cell Tissue Res 376, 113–121 (2019). 30511267 10.1007/s00441-018-2963-2

[58] Zhou Q. Novel Insights Into MALAT1 Function as a MicroRNA Sponge in NSCLC. Front Oncol 11, 758653 (2021). 34778078 10.3389/fonc.2021.758653 PMC8578859

[59] McCown P.J., Wang M.C., Jaeger L. & Brown J.A. Secondary Structural Model of Human MALAT1 Reveals Multiple Structure-Function Relationships. Int J Mol Sci 20(2019). 10.3390/ijms20225610 PMC6888369 31717552

[60] Bouxsein M.L. Guidelines for assessment of bone microstructure in rodents using microcomputed tomography. J Bone Miner Res 25, 1468–86 (2010). 20533309 10.1002/jbmr.141

[61] Deng Z. Def6 regulates endogenous type-I interferon responses in osteoblasts and suppresses osteogenesis. Elife 9(2020). 10.7554/eLife.59659 PMC7771961 33373293

[62] Xia Y. TGFbeta reprograms TNF stimulation of macrophages towards a non-canonical pathway driving inflammatory osteoclastogenesis. Nat Commun 13, 3920 (2022). 35798734 10.1038/s41467-022-31475-1 PMC9263175

[63] Gosset M., Berenbaum F., Thirion S. & Jacques C. Primary culture and phenotyping of murine chondrocytes. Nat Protoc 3, 1253–60 (2008). 18714293 10.1038/nprot.2008.95

[64] Chu C., Quinn J. & Chang H.Y. Chromatin isolation by RNA purification (ChIRP). J Vis Exp (2012). 10.3791/3912 PMC3460573 22472705

[65] West J.A. The long noncoding RNAs NEAT1 and MALAT1 bind active chromatin sites. Mol Cell 55, 791–802 (2014). 25155612 10.1016/j.molcel.2014.07.012 PMC4428586

[66] Stuart T. Comprehensive Integration of Single-Cell Data. Cell 177, 1888–1902 e21 (2019). 31178118 10.1016/j.cell.2019.05.031 PMC6687398

[67] Inoue K. Bone marrow Adipoq-lineage progenitors are a major cellular source of M-CSF that dominates bone marrow macrophage development, osteoclastogenesis, and bone mass. Elife 12(2023). 10.7554/eLife.82118 PMC10005769 36779851

[68] Direder M. The transcriptional profile of keloidal Schwann cells. Exp Mol Med 54, 1886–1900 (2022). 36333467 10.1038/s12276-022-00874-1 PMC9722693

[69] Herkt S.C. Protein arginine methyltransferase 6 controls erythroid gene expression and differentiation of human CD34(+) progenitor cells. Haematologica 103, 18–29 (2018). 29025910 10.3324/haematol.2017.174516 PMC5777187

[70] Locascio J.J. Association between alpha-synuclein blood transcripts and early, neuroimaging-supported Parkinson’s disease. Brain 138, 2659–71 (2015). 26220939 10.1093/brain/awv202 PMC4643625

[71] Jain V. Single Cell RNA-Seq Analysis of Human Red Cells. Front Physiol 13, 828700 (2022). 35514346 10.3389/fphys.2022.828700 PMC9065680

[72] Paul F. Transcriptional Heterogeneity and Lineage Commitment in Myeloid Progenitors. Cell 163, 1663–77 (2015). 26627738 10.1016/j.cell.2015.11.013

[73] Ito Y., Nakahara F., Kagoya Y. & Kurokawa M. CD62L expression level determines the cell fate of myeloid progenitors. Stem Cell Reports 16, 2871–2886 (2021). 34798065 10.1016/j.stemcr.2021.10.012 PMC8693656

