# Supplementary material for: Long non-coding RNA Malat1 fine-tunes bone homeostasis and repair by orchestrating cellular crosstalk and the β-catenin-OPG/Jagged1 pathway
Source: Res Sq. 2024 Oct 11:rs.3.rs-3793919. Originally published 2023 Dec 28. Preprint. [Version 3] doi: 10.21203/rs.3.rs-3793919/v3 (PMC10793491; doi:10.21203/rs.3.rs-3793919/v3)

Supplementary figure legends

Supplementary Fig. 1 Malat1 deficiency does not affect mouse body weight and cortical bone. (**a**) Body weight of 12-week-old male WT and *Malat1*$^{-/-}$ littermates. n=6/ group. (**b**) µCT images and bone morphometric analysis of cortical bone of the mid-shaft femurs isolated from 12-week-old male WT and *Malat1*$^{-/-}$ littermates. n = 6/group.

Supplementary Fig. 2 Malat1 deficiency in *Malat1*$^{\Delta Ocn}$ mice does not affect body weight and cortical bone. (**a**) Body weight of 12-week-old male *Malat1*$^{f/f}$ and *Malat1*$^{\Delta Ocn}$ littermate mice. n=5/group. (**b**) µCT images and bone morphometric analysis of cortical bone of the mid-shaft femurs isolated from 12-week-old male *Malat1*$^{f/f}$ and *Malat1*$^{\Delta Ocn}$ littermates. n = 5/group.

Supplementary Fig. 3 Murine and human Malat1 probe sequences and their complementary Malat1 sequences.

Supplementary Fig. 4 Immunofluorescence staining of β-catenin. Immunofluorescence staining of β-catenin (green) translocation into nuclei with or without Wnt3a treatment for 1h in calvarial osteoblasts isolated from the WT and *Malat1*$^{-/-}$ mice. Arrows: nuclear β-catenin. Scale bar: 50 µm.

Supplementary Fig. 5 Bioinformatic analysis of the scRNAseq dataset GSE128423. (a) A clustering tree of the scRNAseq dataset (GSE128423) across resolutions. **(b)** Dot plots of several typical marker gene expression for each cell type across the listed scRNAseq clusters. Cell clusters are listed on the y-axis. Features are listed along the x-axis. Dot size reflects the percentage of cells in a cluster expressing each gene. Dot color reflects scaled average gene expression level as indicated by the legend.

Supplementary Fig. 6 Verification of the cellular characteristics of the primary chondrocytes isolated from mouse knees. (**a**) Alcian blue staining of primary chondrocytes isolated from the WT and *Malat1*$^{-/-}$ littermate mice. (**b**) Immunofluorescence staining of aggrecan (green) of primary chondrocytes derived from WT and *Malat1*$^{-/-}$ mice. Nuclei were counterstained with DAPI (blue). Scale bar: a 100 µm, b 50 µm.

Supplementary Fig. 1

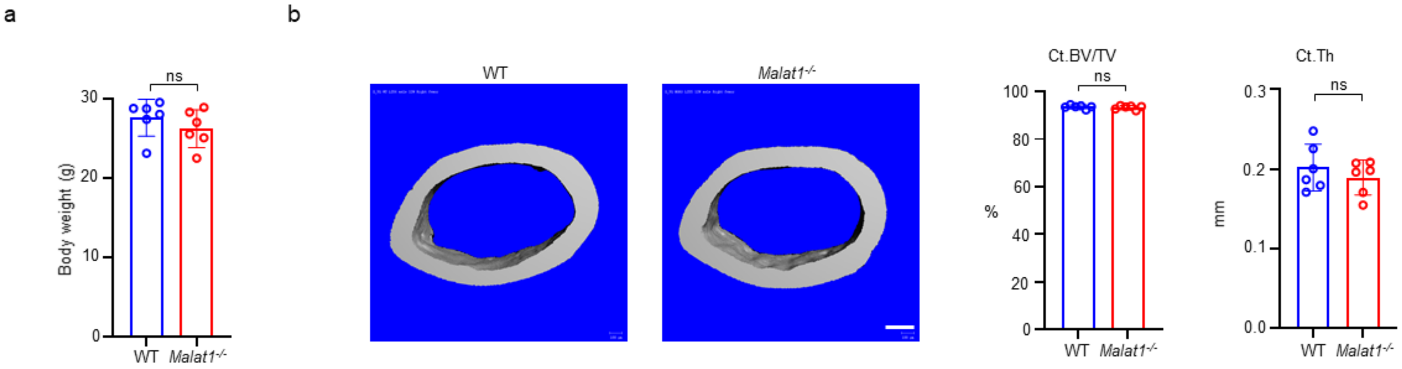

Supplementary Fig. 2

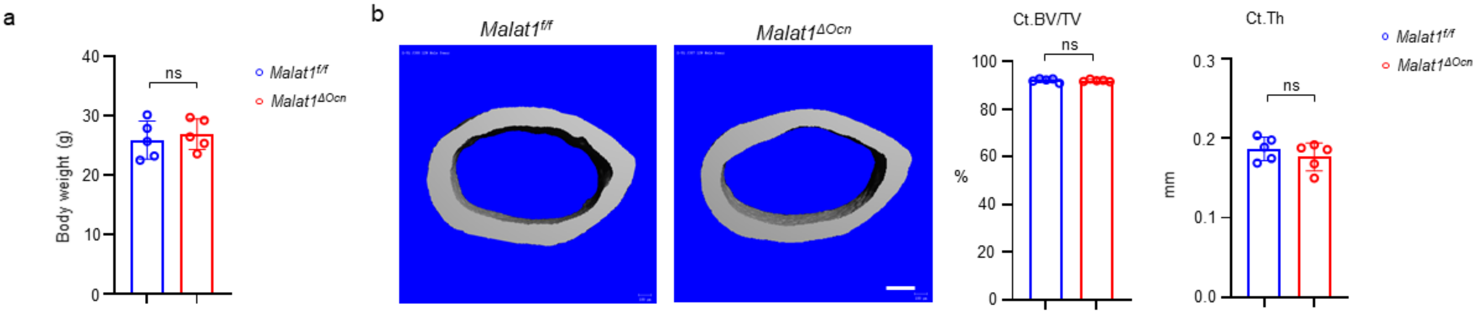

Supplementary Fig. 3

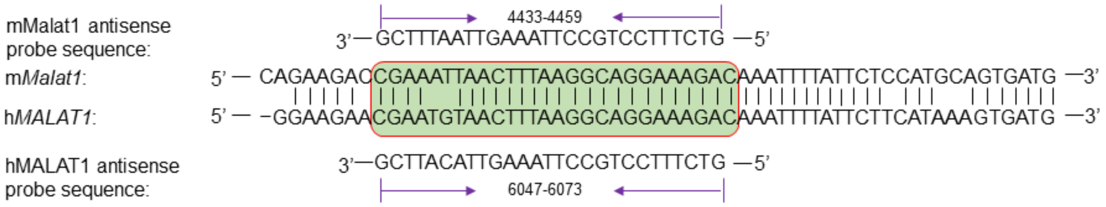

Note: The sequences listed are from RefSeq RNA annotations: NR_002847.3 for mMalat1 and NR_002819.4 for hMALAT1.

Supplementary Fig. 4

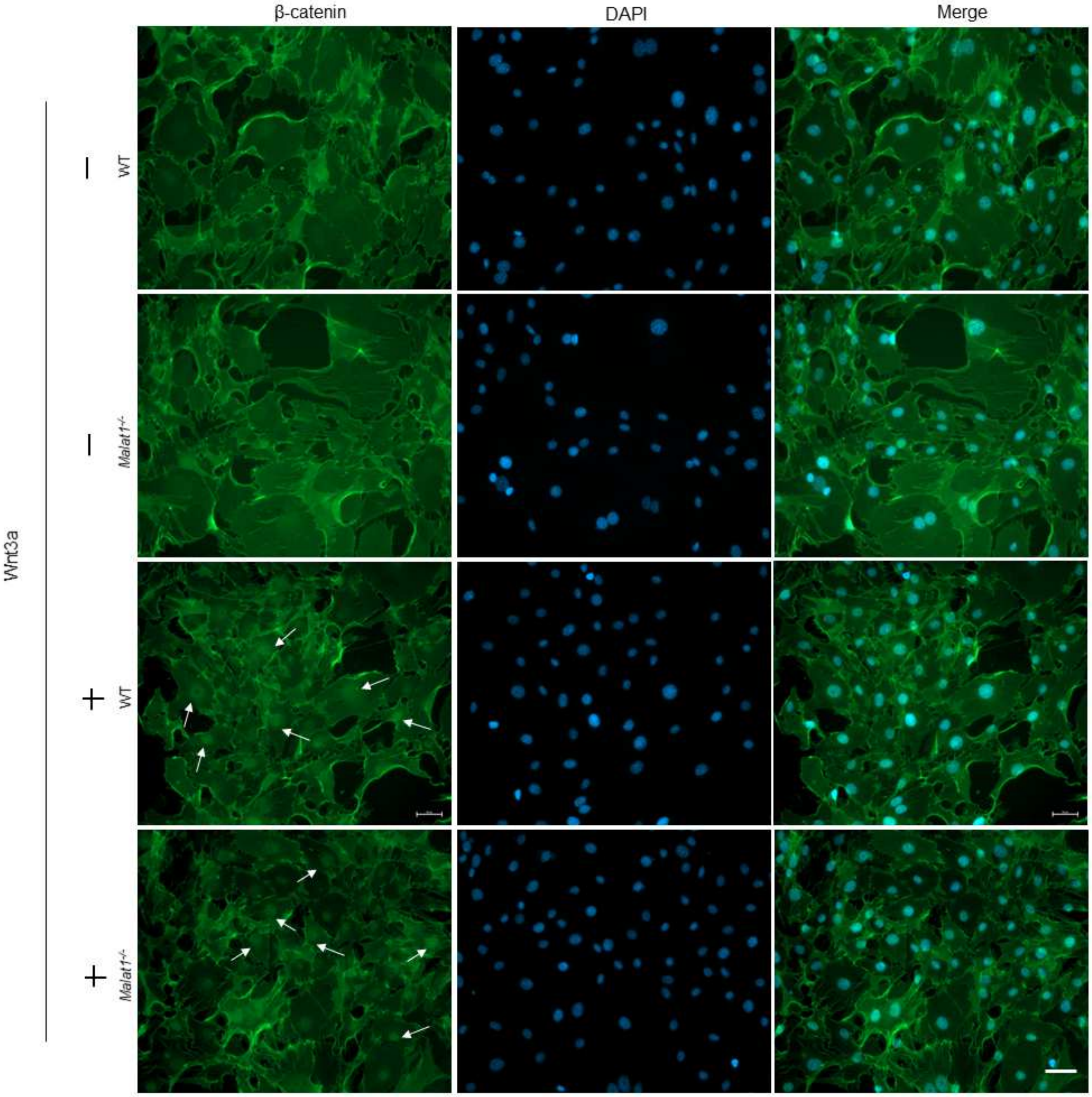

Supplementary Fig. 5

a

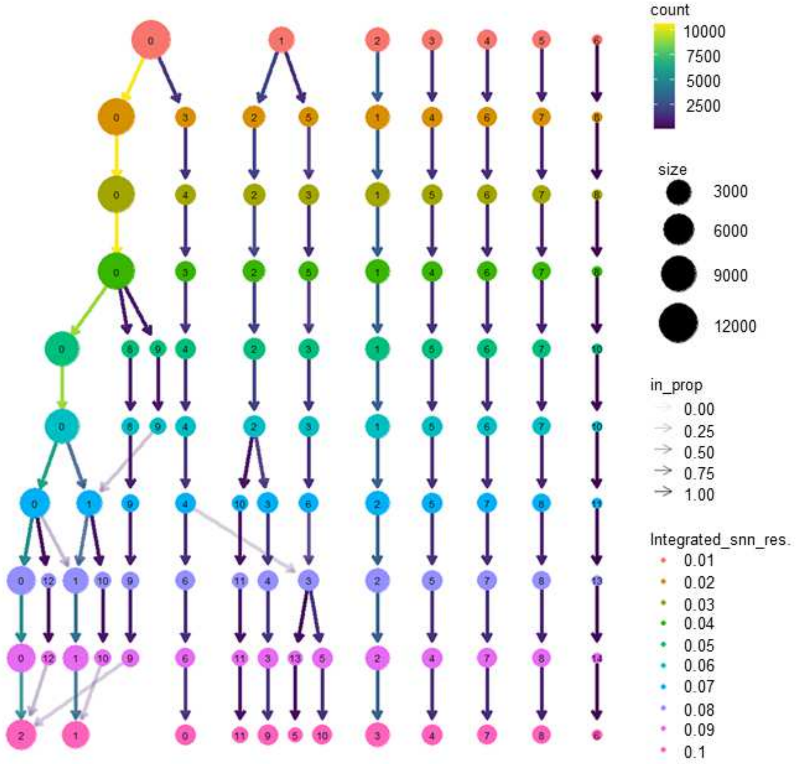

b

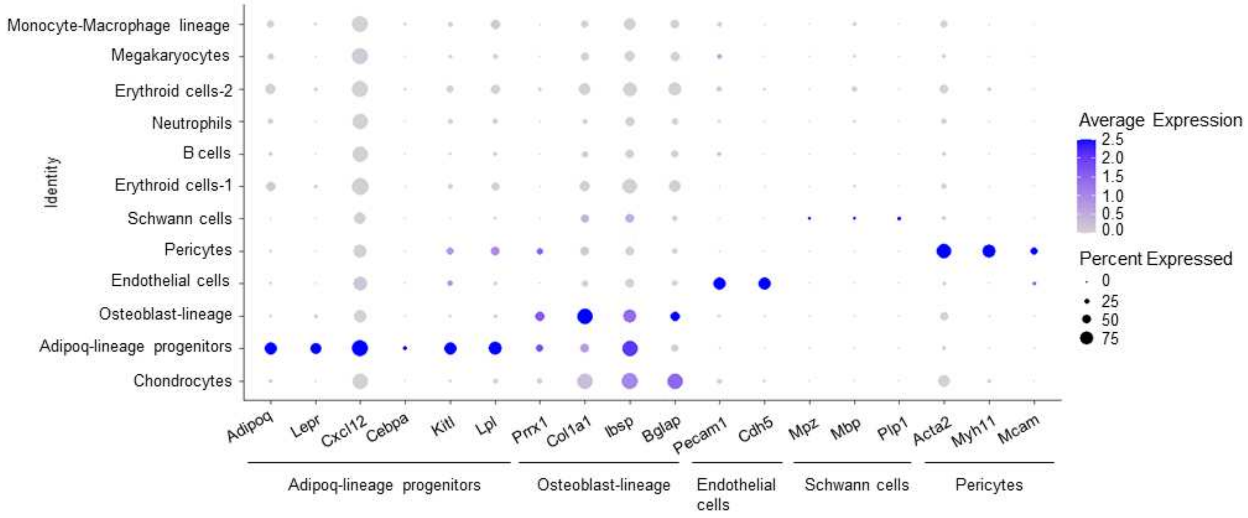

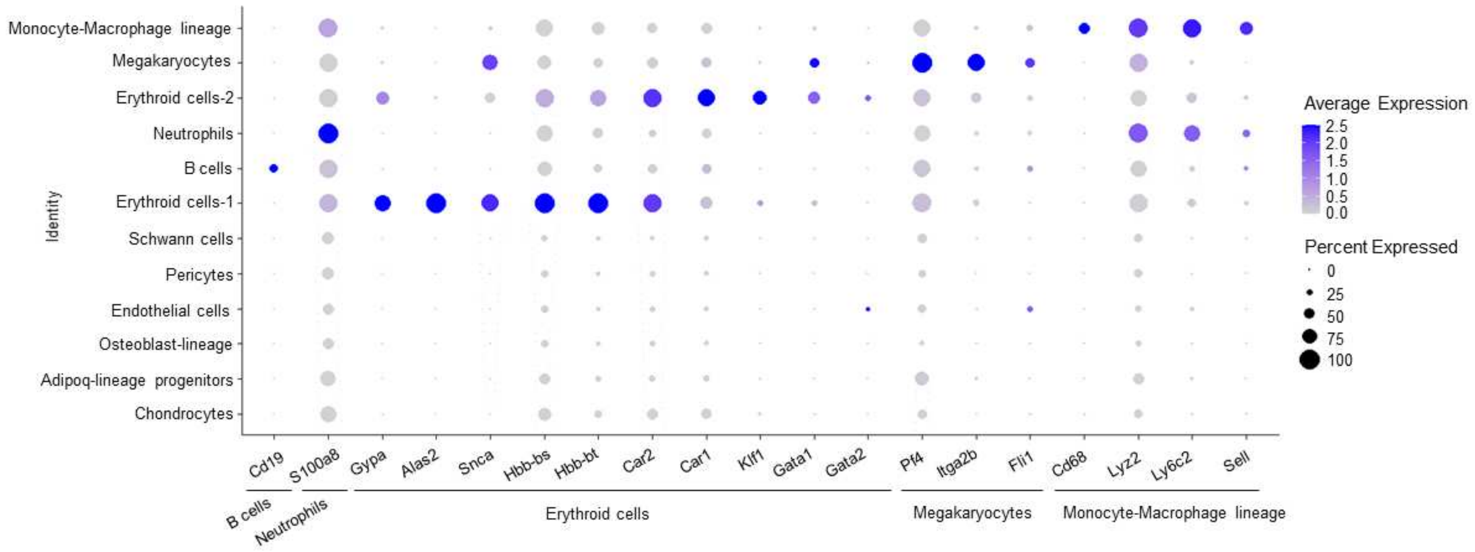

Supplementary Fig. 6

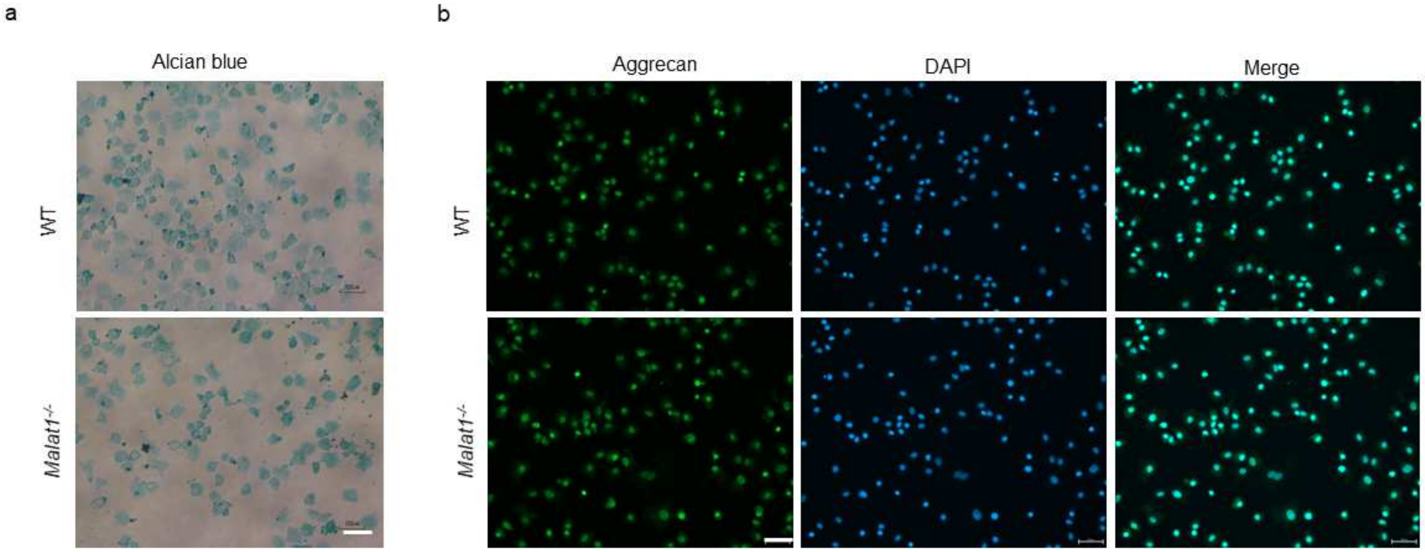

Supplement: Supplement 1 [file NIHPPrs3793919v3-supplement-1.pdf]
